# Longitudinal Hierarchy Co_3_O_4_ Mesocrystals with High-dense Exposure Facets and Anisotropic Interfaces for Direct-Ethanol Fuel Cells

**DOI:** 10.1038/srep24330

**Published:** 2016-04-14

**Authors:** Diab Hassen, Sherif A. El-Safty, Koichi Tsuchiya, Abhijit Chatterjee, Ahmed Elmarakbi, Mohamed. A. Shenashen, Masaru Sakai

**Affiliations:** 1 National Institute for Materials Science (NIMS), Research Center for Strategic Materials, 1-2-1 Sengen, Tsukuba-shi, Ibaraki-ken, 305-0047, Japan; 2 Graduate School of Advanced Science and Engineering, Waseda University, 3-4-1 Okubo, Shinjuku-Ku, Tokyo, 169-8555, Japan; 3 Dassault System Biovia K.K., Materials Science Enterprise, ThinkPark Tower, 2-1-1 Osaki, Shinagawa-ku, Tokyo, 141-6020, Japan; 4 Department of Computing, Engineering and Technology, University of Sunderland, Edinburgh Building, Chester Road, Sunderland, SR1 3SD, United Kingdom; 5 Center for Research in Isotopes and Environmental Dynamics, Tsukuba University, 1-1-1 Tennodai, Tsukuba, Ibaraki, 305-8572, Japan

## Abstract

Novel electrodes are needed for direct ethanol fuel cells with improved quality. Hierarchical engineering can produce catalysts composed of mesocrystals with many exposed active planes and multi-diffused voids. Here we report a simple, one-pot, hydrothermal method for fabricating Co_3_O_4_/carbon/substrate electrodes that provides control over the catalyst mesocrystal morphology (i.e., corn tubercle pellets or banana clusters oriented along nanotube domains, or layered lamina or multiple cantilevered sheets). These morphologies afforded catalysts with a high density of exposed active facets, a diverse range of mesopores in the cage interior, a window architecture, and vertical alignment to the substrate, which improved efficiency in an ethanol electrooxidation reaction compared with a conventional platinum/carbon electrode. On the atomic scale, the longitudinally aligned architecture of the Co_3_O_4_ mesocrystals resulted in exposed low- and high-index single and interface surfaces that had improved electron transport and diffusion compared with currently used electrodes.

Fuel cells are a promising means of powering electric vehicles because they provide high power densities at low cost with zero emissions^1^,^2^,^3^,^4^,^5^,^6^,^7^. Direct ethanol fuel cells have several advantages over other types of fuel cell because they have a high power density, have high efficiency, and are relatively eco-friendly^8^,^9^. Tailored nano-object design and surface morphologies may provide electrode catalysts with improved electroactive site accessibility, leading to preferential surface coverage of ethanol molecules, shorter ion/electron diffusion pathways, better mass transfer of reactants through the nanoscale structure, and faster or reversible kinetics in the ethanol electrooxidation reaction^10^,^11^. However, the effects of catalyst morphology, in terms of anisotropy, density of exposed active sites, and density of multi-diffusive voids in the direction of the longitudinal axis, on the ethanol electrooxidation reaction remains unknown^12^.

Nanosized transition metal oxides and their hybrids are considered among the most promising choices in electrocatalysis because of their unique characteristics in terms of high surface area, easy preparation at low cost, environmental friendliness, structure flexibility, and excellent mechanical and chemical properties^13^,^14^. Nanoporous non-precious metal oxide-based catalysts and electrodes also receive great attention in fuel cell technologies because of their good catalytic activity and enhanced tolerance to organic molecule poisoning^15^. Carbon (C) materials, such as C nanotubes (C-NTs), C black, and graphene (g-C), act as excellent catalyst supports in electrochemical applications owing to their unique characteristics of high electrical conductivity, high specific surface area, good chemical stability, and good corrosion resistance^16^. The synergetic advantages of C materials integrate a fast trigger response to the target on the fabricated surface electrodes. The development of metal oxide/C nanocomposites with controlled morphologies and geometries opens up exciting new avenues for improving the electrochemical performance of pristine metal oxide catalysts^17^.

Considerable efforts have been made in developing single-crystal cobalt (II, III) oxide (Co_3_O_4_) architectures with unique nanoscale shapes (e.g., sheets, cubes, rods, and belts) and predominantly exposed surfaces in the {111}, {100}, {110}, and {112} planes^18^,^19^,^20^,^21^,^22^,^23^,^24^,^25^,^26^. For example, the cubic, closely packed structure of spinel Co_3_O_4_ mesocrystals, which is composed of Co^3+^ and Co^2+^ ions occupying octahedral and tetrahedral coordination sites, plays a significant role in the material’s high catalytic performance^24^. In particular, the exposed {110} plane of Co_3_O_4_ NCs is more catalytically active for carbon monoxide oxidation than are the {100} and {111} planes because of the abundance of rich Co^3+^ active sites in the {110} plane^24^. The exposed surfaces of Co_3_O_4_ NCs have oxygen reduction activities in the order {111} > {100} ≫ {110}, which is consistent with the density of highly exposed Co^2+^ active sites in each plane^15^. Furthermore, the high-surface-energy {112} facets of Co_3_O_4_ NCs have been shown to perform better during methane combustion than the {001} and {011} planes^25^.

Assessing the surface-dependent characteristics of Co_3_O_4_ mesocrystals in terms of high-energy surfaces and density of exposed facets associated with low- and high-index single and interface planes is important when considering their use in ethanol electrooxidation. The scalable, low-cost, direct growth of homogeneous nanostructures comprising multi-component composites along the longitudinal axis of conductive substrates may provide favorable synergetic properties and competitive advantages over currently used electrodes in terms of enhanced conductivity, better structural stability and flexibility, superior electron-collection efficiency, and richness of electroactive sites^27^,^28^,^29^,^30^,^31^.

Here we report a simple, one-pot, hydrothermal method of synthesizing various electrode architectures along the longitudinal axis by using multi-component carbon/Co_3_O_4_/substrate layers. This method provided versatile control over the production of a variety of anisotropic morphological Co_3_O_4_ architectures with low- and high-index single and interface planes with multi-diffusive mesocage cavities and windows. Our developed electrodes had improved catalytic efficiency for ethanol electrooxidation compared with a conventional platinum/carbon (Pt/C) electrode, and therefore offers a potentially effective solution to the issues surrounding the practical application of direct ethanol fuel cells.

## Results and Discussion

**Figure 1:**
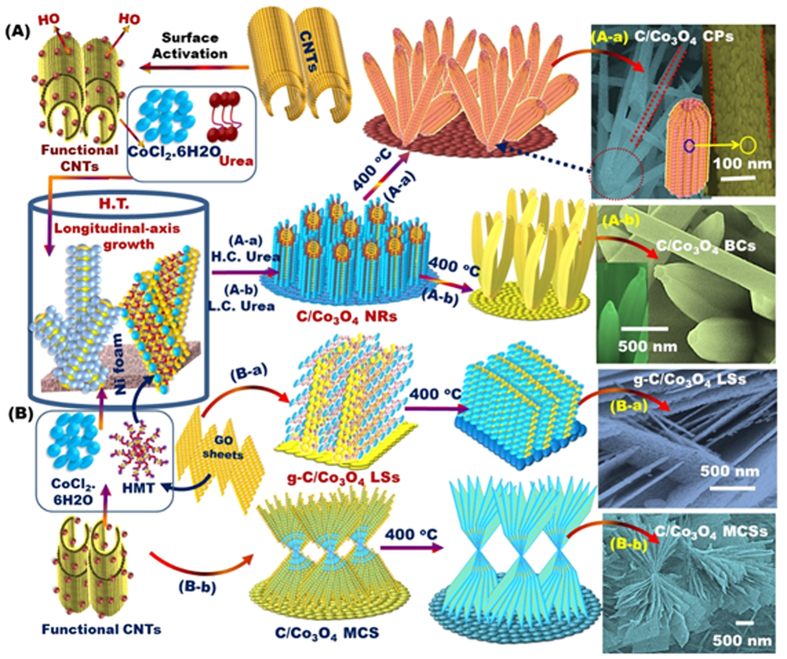
(**A,B**) Schematic of the one-pot hydrothermal synthesis of electrode fabrics using hierarchical Co_3_O_4_ nanocrystals formed under the controlled engineering of surface morphologies along the longitudinal scales of corn-pellets (CPs) (**A**-a) and banana-clusters (BCs) (**A**-b) in nanorod dominates and nanolayered stacking-sheets (**B**-a) and multiple cantilevered sheet- (MCS) (**B**-b) based surface morphologies. The counterparts of C-NTs and graphene (g-**C**) were used as conducting supports. The C-NTs were employed to fabricate C-NTs/C_3_O_4_ CPs, C-NTs/C_3_O_4_ BCs, and C-NTs/C_3_O_4_ MCSs, whereas graphene was used to synthesize g-C/C_3_O_4_ mesocrystals. The CP-, and BC-NR cylindrical roller sizes vary from the sub-nanometer scale to over a few nanometers, depending on the dose of urea added to the synthetic composition and the number of the construction block units along and around the C-axis (**A**-a,**A**-b).

Figure 1 shows our simple, one-pot, hydrothermal method for synthesizing self-supported electrodes with novel morphological architectures that are comparable with previously reported nanostructures (Table S1). The overall structure of the electrodes was [carbon support/Co_3_O_4_ or cobalt (II) oxide (CoO)/substrate], where the carbon support was either functionalized multi-walled carbon nanotubes (C-NTs) or graphene sheets (g-C), and the substrate was 3D porous Ni foam (3D PNi) (see Experimental Section & Supplementary Sections S1–S8 in the Supplementary Information). The technical advantages of the proposed method are its simplicity, scalability, and low-cost operation. Furthermore, the proposed method allows the synthesis of Co_3_O_4_ or CoO mesocrystal architectures along the longitudinal plane with surface morphologies oriented vertically away from the substrate in the form of corn tubercle pellets (CPs) or banana clusters (BCs) oriented in nanorod (NR) domains, lamina sheets (LSs), or multiple cantilevered sheets (MCSs)(Supplementary Figures S1–S4).

Co_3_O_4_ mesocrystals with morphologies of CP-NR, BC-NR, LS, or MCS were well dispersed interiorly and oriented vertically away from the 3D PNi support. Monodispersed particles were attached to the functional core of the *c*-axis, on which the novel, smart CP, BC, LS, and MCS mesocrystal morphologies were formed. Field emission scanning electron microscopy revealed that our method afforded mesocrystal morphologies with the following key architectural features (Supplementary Figures S1–S4):

1. Dense aggregation and constituent attachment of Co_3_O_4_ CP-NRs and BC-NRs along the longitudinal axis with an axial band of CP-NRs or BC-NRs closely packed around a central focal point, leading to the formation of nanoforests comprising structures that resembled giant bamboo trees.
2. The size of the CP-NRs and BC-NRs in the “bamboo trees” decreased with increasing distance from the central focal point and arched along the *c*-axis, forming a sharp-edged, convex, needle-like NR architecture with a nanoscale window running internally along the *c*-axis. This dominant needle-shaped end top surface enabled rapid electron transport by maximizing the diffusion of trigger pulse electrons during the catalytic reactions.
3. The randomly oriented C-NTs with an intrinsic tubular structure (core) greatly enhanced the longitudinal growth of CP and BC mesocrystals and their crystal surfaces in a vertical fashion along the *c*-axis because of the interaction between the carboxylic groups onto the functional C-NTs (Scheme 1) and the positively charged Co^2+^ ions^32^. In turn, the penetrating growth of the Co_3_O_4_ mesocrystals among the stacking GO layers strongly indicated the formation of LS mesocrystals. The vertical growth of the g-C/LS nanolayered stacking sheets was due to the integrative contribution of both electrostatic interaction and Van der Waals forces.
4. Addition of the surfactant hexamethylenetetramine during catalyst synthesis promoted the formation of dense, vertical lamina aggregates of LSs and MCSs that had distal, non-stacked layers bordered by spacer micro-, meso-, and macro-globule pores, as shown by N_2_ isothermal profiling (Supplementary Section S7 and Supplementary Figure S7). Therefore, the growth along the vertical axis of the LSs and MCSs originated from the directional growth along the longitudinal axis.
5. Use of g-C as the carbon support caused the LSs to be discretely stacked with regular spacing (100 nm) or to not stack at all. This multilayered LS structure along the longitudinal axis may be responsible for the efficient catalytic activity observed compared with that of complex MCS architectures with inverse opal multilayers.
6. The use of C-NTs, cobalt (II) chloride (CoCl_2_), and hexamethylenetetramine resulted in the preferential axial arrangement of flattened double-helix bands running in opposite directions and production of multiple-cantilevered sheets along the longitudinal axis of the C-NT domains. Each individual flattened sheet exhibited dynamic flexibility in its curvature in the double-concave direction along each edge (Supplementary Figures S3 and S4).

**Figure 2:**
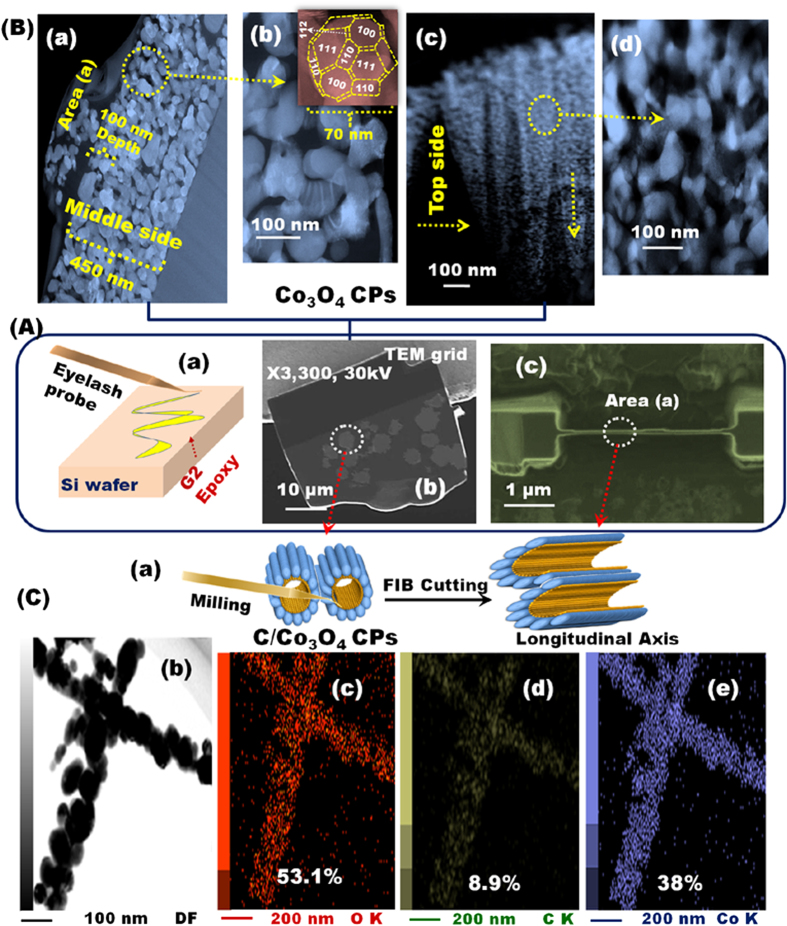
(**A**-a–**A**-c,**B**-a–**B**-d,**C**-b–**C**-e) HAADF-STEM micrographs of single Co_3_O_4_ CPs and C-NT/Co_3_O_4_ CPs after sample operation of the interior structure of the microtomed samples using the FIB system. (**A**-a–**A**-c) Schematic representation of the sample preparation sequence for the FIB system and (**B**-a–**B**-d) the corresponding low-magnification SEM images. The STEM–bright/dark field images of the pristine Co_3_O_4_ CPs after subjecting to the FIB system at the middle section (**B**-a–**B**-b) and along the top side (**B**-a,**B**-d) of the microtomed sample. With the CP resemblance in the NRs (**B**-c), many protruded particles arched in the pellet bunches and massive constructions of polyhedron mesocrystal facets were shaped like a giant fortress with windows throughout the NR columns. The inset displays a high-magnification image of a single polyhedron of 70 nm. (**C**-b–**C**-e) STEM and STEM–EDS mapping of C-NT/Co_3_O_4_ CPs microtomed sample after FIB investigation. (**C**-b) STEM–dark field image of the microtomed sample. (**C**-c–**C**-e) STEM–EDS mapping showing the elemental composition of oxygen (c), carbon (d), and cobalt (e) and the corresponding percentage of each element.

The vertical alignment of the CP-NR, BC-NR, LS or MCS mesocrystal architectures along the longitudinal plane remains a topic of interest with regard to ethanol oxidation reactions. To investigate the internal concentric arrangement and orientation of the constituent units inside these structural morphologies, we conducted etching experiments by using a focused-ion beam milling setup to etch specimens parallel to the longitudinal axis to a thickness of 100 nm (Fig. 2A,B) Supplementary Section S9). A focused-ion beam system for fabricating thin specimens was integrated into our high angular annular dark-field (HAADF)–scanning/transmission electron microscope system (STEM) (Fig. 2 and Supplementary Figure S9) and STEM–energy dispersive X-ray spectroscopy (EDS) mapping analysis system (Fig. 2C).

Figure 2B-a–1B-d shows the interior structure of the Co_3_O_4_ CP morphology at a depth of 100 nm and at different locations along the longitudinal axis of the NRs. The concrete arrangements of the protruding pellets on these corn tubercle surfaces and surface in homogeneity readily sit on the apex of C-NTs covered by numerous oriented tiny tubercles. The internal atomic arrangement of the nanoforests was also well dispersed longitudinally (STEM–EDS mapping, Fig. 2C). The CPs in the Co_3_O_4_ NR skin were ribbed and vertically configured to the longitudinal axis grooves (aligned parallel to the main direction of NR mesocrystal projection), which created canals and window spaces for electron diffusion and transport (i.e., upward and downward movements; Fig. 2 and Supplementary Figure S9). The STEM–EDS observations of the selected area (Fig. 2Cb–Ce) confirm that the hybrid composites are mainly composed of Co, O, and C. The elemental mapping of the microtomed C/Co_3_O_4_ CP hybrids indicates that the trunk of the micromoted nanocorn tubercles is mainly composed of Co, and the core of the nanocorn tubercles is composed of homogeneous C nanowires. This result demonstrates that C-NT acts similar to a scaffold for the growth of metal oxide NPs. The distribution of each element in the CP hybrid composites is highly consistent with the selected area of nanocorn tubercles. In accordance with the HAADF and STEM-EDS analyses, the observed atomic content of C is basically from the hybrid catalyst, as evidenced from the existence of C-NTs along the nanocorn tubercles.

**Figure 3:**
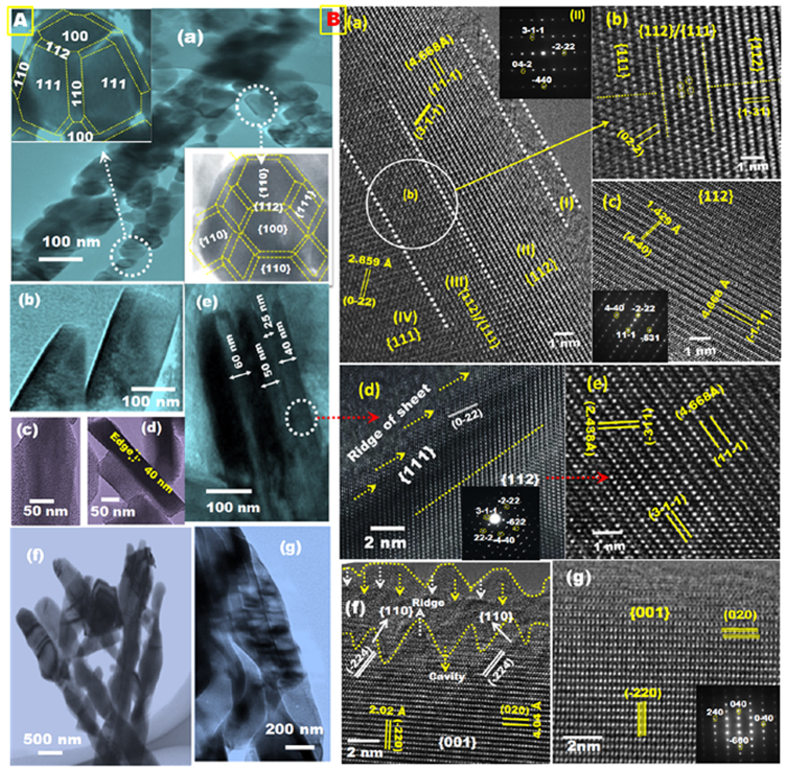
(**A-**a**,A-**b**–A-**e**,A-**f**–A-**g**,B-**a**–B-**c**,B-**d**–B-**e**,B-**f**–B-**g) low and high magnification HAADF-STEM micrographs of C-NT or g-C/Co_3_O_4_/3D PNi electrodes, (**A**-a,**A**-b–**A**-e,**A**-f–**A**-g) low magnification TEM views of C-NT/Co_3_O_4_ CP, g-C/Co_3_O_4_ LSs and C-NT/Co_3_O_4_ BCs/3D PNi respectively, (**A**-a- inserts) illustrating the exposed surface facets of non well-defined polyhedrons C-NT/Co_3_O_4_ CPs mesocrystals, (**B**-a–**B**-c,**B**-d–**B**-e,**B**-f–**B**-g) high resolution HAADF-STEM images show the corresponding atomic structure of the developed nanostructures recorded along the dominant {112} (**B**-c,**B**-e), and {001} (**B**-g) surface planes and {111/112} (**B**-a,**B**-b,**B**-d) and {110/001} (**B**-f) interfaces, and (**B**-a,**B**-c,**B**-d,**B**-g)-inserts show the related electron diffraction patterns viewed down the {111}, {112}, {111}, and {001} respectively.

HAADF–STEM microscopy revealed the morphologies along the longitudinal axes of the NRs (i.e., C/Co_3_O_4_ CPs and C/Co_3_O_4_ BCs) and nanosheets (i.e., C/Co_3_O_4_ MCSs and g-C/Co_3_O_4_ LSs) (Fig. 3A). Individual CP domains exhibited a marked side-to-side anisotropic geometry with well-distributed atomic surface active sites (Fig. 3A-a). High-resolution HAADF–STEM microscopy of 50-nm particles (Fig. 3A-a, inset) revealed the proposed mesocrystal model of 50-facet polyhedrons. The 50-facet Co_3_O_4_ CPs, as well as other non-well-defined polyhedral mesocrystals, grew mainly along the low-index {100} and {111} planes together with some high-index {112} planes and other poorly developed facets. Twenty-four exposed high-index {112} planes had grown axially around each {100} and {111} planes of the 50-facet polyhedrons. Increasing the number of high-index planes with a high surface energy and the number of polyhedron CPs oriented longitudinally within the NR morphology are key to improving the performance of the electrodes for ethanol oxidation.

Side-view HAADF–STEM microscopy of the Co_3_O_4_ MCSs revealed lamina sheets that had been flattened during reassembly and had rough surfaces containing pores aligned perpendicular to the longitudinal axis (Fig. 3A-b,c). Top-view HAADF–STEM microscopy of the Co_3_O_4_ LSs revealed a series of non-stacked, well-oriented, vertical lamina sheets. Nanoscale (40–60 nm) edges of the lamina sheets were located laterally along the longitudinal axis (Fig. 3A-d,e). HAADF–STEM microscopy of the Co_3_O_4_ BCs revealed that they represent the visual architecture of an actual banana cluster with structures built upon one-dimensional NRs and control boundary-layer skin as tunnel-line nanopatterns, dense particles, and smooth surfaces (Fig. 3A-f,g).

High-resolution HAADF–STEM micrographs (Fig. 3B) and the corresponding EDS patterns (Fig. 3B, insets) taken along {111}/{112} (Fig. 3B-a,b) and the dominant {112} (Fig. 3B-c), {111} (Fig. 3B-a,d), {112} (Fig. 3B-e), {110}/{001} (Fig. 3B-f), and {001} (Fig. 3B-f,g) planes further revealed the structures of single-crystals of the Co_3_O_4_ CPs, LSs, MCSs, and BCs, respectively. Mesocrystal growth in as-prepared samples of Co(OH)_x_(CO_3_)_0.5_∙0.11H_2_O with CP-NR, BC-NR, LS, or MCS morphologies primarily produced longitudinally aligned facets exposed along the {001} plane, (Supplementary Section S10). High-temperature treatment of the CP-NRs, BC-NRs, MCSs, and LSs may strongly influence the atomic configuration and renucleation within the architectures, which would enable sustained relaxation of the atoms around the dominant {001} facet to produce the high surface energy {112} and {111}/{112} planes of the CPs, MCSs, and LSs (Fig. 3B-a–e), and the {110}/{001} plane (Fig. 3B-f,g) of the BCs.

The axial orientation change of the crystal planes along the {001} direction under treatment led to the remarkable formation of {111}/{112} interface planes between the {112} and {111} planes of the Co_3_O_4_ CPs. A multi-step-terrace topography over the ridges of the nanosheets also caused {111}/{112} interface planes to form on the Co_3_O_4_ LSs and MCSs. In turn, a dense construction of BCs was produced on the smooth, flattened lamina surfaces with tightly joined and closely packed basal edges (i.e., flanked border lines) (Figure S10). A protruding surface with a multi-step-terrace topography forming hedge-saw islands with many ridges and cavities along its edges at a depth of 10 nm was also observed (Fig. 3B-f,g). Such an interfacial topography may create an open surface for the diffusion of Co^3+^ adatoms into vacancies in the crystal surface. Consequently, the Co^3+^-enriched {111}/{112} and {110}/{001} interface planes associated with the main topographic facet islands aligned along the {111}, {112}, and {001} planes may form (Supplementary Figure S10). A continuous electron flow may then be produced parallel to the longitudinal axes of the nanoforests.

**Figure 4:**
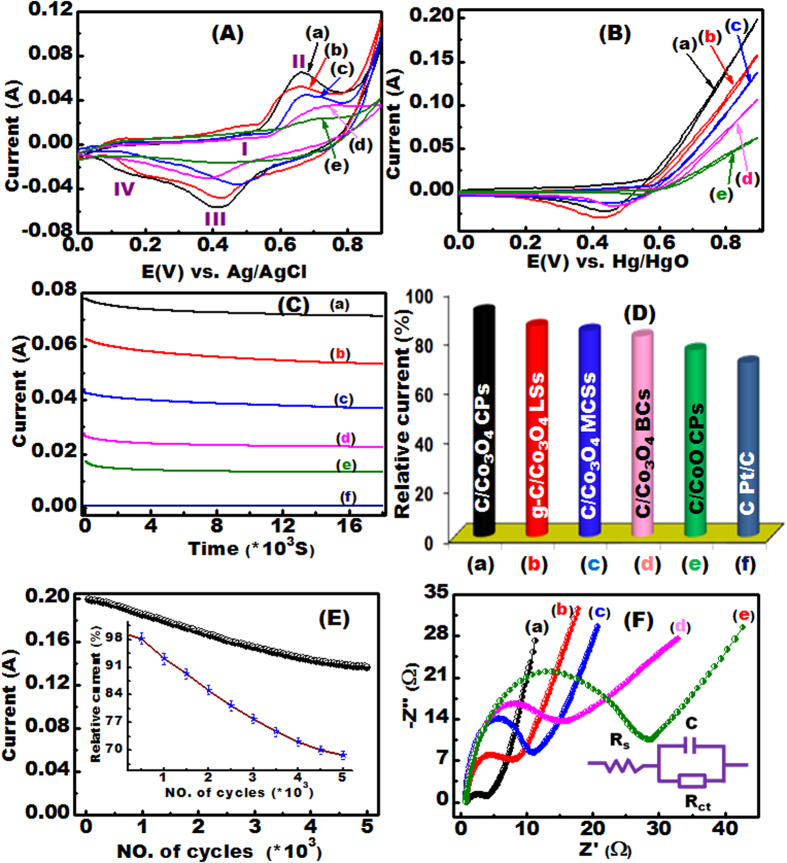
(**A–F**) Electrochemical characterization of self-supported electrodes recorded in 0.5 M NaOH solution at 50 mV s^−1^ under room temperature. (**A**) CV profiles of the applied electrodes recorded in the absence of ethanol. (**B**) CV spectra of the proposed electrodes collected in the presence of 0.5 M ethanol. (**C**) Current–time relationship of the electrodes obtained by CA technique measured in 0.5 M ethanol at a fixed applied potential of 0.7 V for 18,000 s and compared with the commercial Pt/C electrode (Fig. 5C-curve f). (**D**) Relative current of the investigated electrodes measured at the end of the current–time analyses. (**E**) Net current of the C-NT/Co_3_O_4_ CPs/3D PNi electrode as a function of the effective number of cycles; the inset (g) shows the plot of the relative current and the corresponding number of cycles used. (**F**) EIS profiles of the electrodes recorded in the presence of 0.5 M C_2_H_5_OH at 5 mV amplitude. (**F**, inset) The solution resistance (Rs) and charge-transfer resistance (Rct) in the electrode and at the electrode–electrolyte interface, respectively, as well as the redox capacitance (**C**), are the equivalent circuit variable described in the Nyquist plots.

Using a catalytic electrochemical assay (Supplementary Section S11–S18), we investigated the effects of the following parameters on ethanol electrooxidation: (i) arrangement and orientation of heterogeneous Co^2+^/Co^3+^ site atomics, (ii) type of nanoforest morphology (CP-NR, BC-NR, LS, or MCS), (iii) mesocrystal orientation with exposed high-index {112} planes, (iv) degree of exposure of interior atomic-scale {111}/{112} interface planes blocks, (v) growth along the longitudinal axis to attain high surface energy and dense Co^3+^ site surfaces, (vi) presence of multi-diffusive pore phases, and (vii) electrode nano-pattern design resulting from the combination of conductive support (C-NT or g-C) and substrate (4D PNi or glass carbon, GC) (Fig. 4 and Supplementary Figures S11–S18).

Electrodes with C-NT or g-C/Co_3_O_4_ or CoO/3D PNi or glassy carbon architectures were used as the anodic electrode in an ethanol electrooxidation reaction (Fig. 4 and Supplementary Information S13–S18). In a non-alcoholic assay, all of the electrodes revealed two sets of redox couples produced from the reversible reactions between Co_3_O_4_ and cobalt oxide hydroxide (CoOOH) (peaks I and IV) and between CoOOH and CoO_2_ (peaks III and/II) (Supplementary Section S11 and Fig. 4A)^33^,^34^. In the ethanol electrochemical assays, no anodic peaks were observed with any of the electrodes, indicating that oxygen evolution overlapped with the ethanol oxidation and that oxygen was evolved at highly positive potentials from 0.6 to 0.9 V (Fig. 4B). In the reverse scan, cathodic peaks from 0.3 to 0.5 V were observed, which were associated mainly with the removal of carbonaceous species (such as carbon monoxide, carbon dioxide, methanoic acid, hydroxymethylene, and methyl methanoate) that were not completely oxidized in the reverse scan and the chemisorption of unknown species onto the electrode (Fig. 4B)^35^,^36^.

Each of the electrodes used in the assay is proposed to form an electroactive mediator CoOOH layer that facilitated ethanol molecule absorption and electron transport, forming the highly active species Co (IV). As shown in Fig. 4B the increase in the current density at 0.9 V follow this order C-NT/Co_3_O_4_ CPs > g-C/Co_3_O_4_ LSs > C-NT/Co_3_O_4_ MCSs > C-NT/Co_3_O_4_ BCs > C-NT/CoO CPs. Current density markedly increased with the addition of ethanol, indicating that the electrodes catalyzed ethanol electrooxidation (Fig. 4A,B and Supplementary Figure S13A,B). Moreover, Co_3_O_4_ mesocrystals, which had catalytically active tetrahedral Co^2+^ and octahedral Co^3+^ sites in exposed high- and low-index planes, showed higher catalytic ethanol electrooxidation activity than did CoO mesocrystals, which had only catalytically active Co^2+^ sites, indicating that surfaces containing both Co^2+^ and Co^3+^ sites have higher catalytic activities than do those with only Co^2+^ sites^37^,^38^,^39^,^40^,^41^.

In the practical use of electrocatalyst in DEFCs, the high-oxidation current density and low onset potential indicate its high catalytic activity and low overpotential. We measure the current density and onset potential, which are the key parameters to indicate the electroactivity and overpotential of the proposed catalysts in DEFC design (Figs 4, S11-F and S13), to evaluate the electrocatalytic efficacy of hierarchy g-C or C-NT/Co_3_O_4_/3D PNi electrodes^13^,^42^. Our finding indicates that the maximum catalytic current density of our proposed electrode C/Co_3_O_4_ CP at 0.9 V (vs. Hg/HgO) is approximately 200 mA cm^−2^, which indicates the superior electroactivity of the C/Co_3_O_4_ CP electrode for EOR. Figure S11F clearly shows that the onset potential for the EOR of the free-supported electrodes follows the order C-NT/Co_3_O_4_ CPs < g-C/Co_3_O_4_ LSs < C-NT/Co_3_O_4_ MCSs < C-NT/Co_3_O_4_ BCs < C-NT/CoO. Among all the proposed electrodes, the C-NT/Co_3_O_4_ CPs exhibit the lowest onset potential and the highest oxidation current density. Moreover, the onset potential observed for the C-NT/Co_3_O_4_ CPs is much lower than those for metal oxide catalysts^42^,^43^ and is analogous to the Co_3_O_4_/NiO, NiCo_2_O_4_, and NiMoO_4_ nanostructures previously reported^16^,^42^,^43^.

To further illustrate the superior electrochemical activity conferred by directly growing C-NT or g-C/Co_3_O_4_ catalysts along the longitudinal axison3D PNi, we also performed ethanol electrooxidation reactions using a C-NT or g-C/Co_3_O_4_/GC electrode and a commercial Pt/C electrode for comparison (Supplementary Figures 13C,D). Compared with the performance of commercial Pt/C electrode and the C-NT or g-CCo_3_O_4_/GC electrode with CP morphology, the performance of C-NT or g-C/Co_3_O_4_/3D PNi or GC electrodes with any morphology was comparable to commercial Pt/C electrode in terms of current density and electrode stability (Fig. 4C,D and Supplementary Figure S13C,D).

To determine the current density and stability of the developed electrodes, current–time relationships were determined by means of chronoamperometric testing in 0.5 M ethanol for 18,000 s with carbon/Co_3_O_4_ or CoO/3D PNi and Pt/C electrodes (Fig. 4C and Supplementary Information S17). Slight decays in the currents of the C-NT or g-C/Co_3_O_4_/3D PNi electrodes indicated superior current stability and resistance to poisoning compared with the C-NT/CoO/3D PNi and Pt/C electrodes (Fig. 4C,D and Supplementary Figure S13C,D). The relative current % at the end of the assessment period (i.e., at t = 18,000 s) decreased in the order C-NT/Co_3_O_4_ CP > g-C/Co_3_O_4_ LS > C-NT/Co_3_O_4_ MCS > C-NT/Co_3_O_4_ BC > C-NT/CoO CP > Pt/C, which is consistent with the change in reaction rate (catalytic rate constant) and diffusion coefficient of the electrodes (Supplementary Table S2 and Supplementary Sections S17–S18). The superior catalytic activity of the C-NT or g-C/Co_3_O_4_ electrodes over the C-NT or g-C/CoO and Pt/C electrodes indicated that manipulation of anisotropic C-NT or g-C/Co_3_O_4_ morphologies along the longitudinal *c*-axis enabled the creation of improved catalytic structural features. Compared with the other electrodes examined, the high-index exposed surfaces and interfaces of C-NT or g-C/Co_3_O_4_ may produce more efficient electron movement over the oxygenated, grooved electrode surface and into Co^3+^ sites via upstream swirls and multi-diffusivity windows.

Optimizing the long-term stability, efficiency, and recyclability of electrodes used in direct ethanol fuel cells remains a challenge. To further assess the durability of the developed electrodes, a set of experimental ethanol electrooxidation reactions was conducted with a C-NT/Co_3_O_4_ CP electrode under continuous potential cycling for 5000 cycles (Fig. 4E and Supplementary Section S19). The electrode retained approximately 68.7% of its original current density after multiple reuse cycles at 0.9 V (≥5000 cycles), which is superior to all other electrodes reported recently (Supplementary Table S3). Notably, after ≥5000 cycles, the reused electrode had retained its highly reactive surfaces (Supplementary Figure S19), demonstrating retention of 71.9% of its current density (Supplementary Figure S16) in the fresh electrolyte of our electrochemical assay. This result suggested that our developed electrodes markedly improved the dead-end workability and recyclability compared with all other electrodes reported recently (see Supplementary Table S3).

We quantitatively investigated the effects of anisotropic morphology and number of surfaces enriched with Co^3+^ sites on charge transfer (i.e., electrical conductivity) within the developed electrodes. Electrodes with architectures of C-NT/Co_3_O_4_ CP, C-NT/Co_3_O_4_ BC, C-NT/Co_3_O_4_ MCS, g-C/Co_3_O_4_ LS, and C-NT/CoO CP were assessed by means of electrochemical impedance spectroscopy using an open circuit potential with a 5 mV amplitude at frequencies from 100 kHz to 0.01 Hz in 0.5 M sodium hydroxide containing 0.5 M ethanol (Fig. 4F-a–e). The impedance Nyquist plots for all of the electrodes had a semicircular shape at high frequencies, which represents the Rct of the electrode design. In addition, they had a straight line at low frequencies, which represents the solution resistance (Rs), charge-transfer resistance (Rct) and the redox capacitive behavior (C) of CoO or Co_3_O_4_^42^ (Fig. 4F-insert). The diameters of the semicircles in the plots increased in the order C-NT/Co_3_O_4_ CP < g-C/Co_3_O_4_ LS < C-NT/Co_3_O_4_ MCS < C-NT/Co_3_O_4_ BC < C-NT/CoO CP.

**Figure 5:**
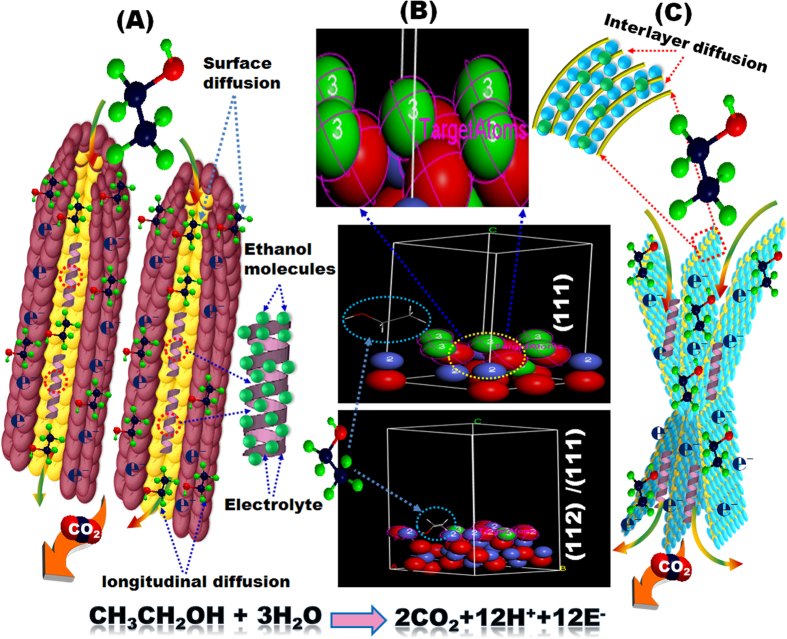
(**A,B**) Schematic representation showing the diffusivity of ethanol molecules and transport of electrons through the hierarchical structures during EOR. (**A**) Longitudinal and surface diffusivity of ethanol molecules along the C-NT/Co_3_O_4_ CPs. (**B**) Ethanol diffusion at the catalytically active sites {111} and {112/111} measured by DFT modeling. (**C**) Interlayer diffusion of ethanol molecules between neighboring nanosheets and Co_3_O_4_ mesocrystals. We propose the optimal electrooxidation of ethanol in multiple steps, resulting in 12 electrons transferred from the electrolyte phase to the binding sites into the hierarchical structures. The electrode patterns with mesocyclinder-open-pore, dense and active site surfaces, longitudinal hub gloves, and distal, and non-stacked layers enable rapid electron movements with little resistance and ensure contact between every mesocrystal coverage surfaces and the electrolyte in a multi-directional accessibility.

**Figure 6:**
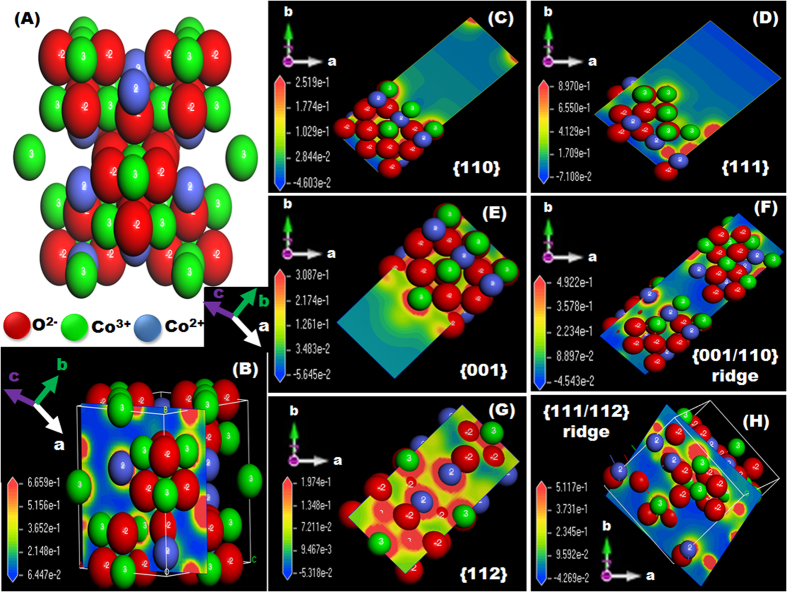
(**A–F**) Theoretical modeling by DFT showing the distribution of EP at the catalytically active centers. (**A**) Optimized structure of C/Co_3_O_4_ CPs indicating the atomic arrangement; the blue, green, and red atoms represent Co^3+^, Co^2+^, and oxygen respectively. The cobalt atoms (Co^3+^ and Co^2+^) are positively charged, whereas the oxygen atoms were negatively charged. (**B**) EP counter over the surface of the suggested structure of C/Co_3_O_4_ CPs. (**C–H**) EP mapping along the exposed surface facets: {110} (**C**), {111} (**D**), {001} (**E**), {001/110} ridge (**F**), {112} (**G**), and (111/112} ridge (**H**).

Importantly, the hierarchal nanoforest architectures created multi-diffusive phases of molecular and electron transport along the catalyst morphology through longitudinal monowindow- and mesocylinder-open-pore architectures (CPs and BCs) (Fig. 5A), vertical inter-layered spaces and interfaces (LSs and MCSs) (Fig. 5C), and catalytically active surfaces and exposure sites (Fig. 5B). In such multi-diffusivity phases, “surface-blocking” by the accumulation of intermediate species can “poison” an electrode surface^44^. In addition, the vertical grooves across the longitudinal axis caused the electron flow to perpetually realign in response to changes in internal and external architectures (Fig. 5A). Thus, the longitudinally oriented surfaces with catalytically exposed sites may play a critical role in improving the kinetics and diffusion of electrons (Fig. 5B). The proposed model (Fig. 5B) takes account of electrode surfaces composed of Co^2+^, Co^3+^, and O^2−^ atoms stacked alternately along the *c*-axis that become dense at the upper area of the coverage surfaces of the {111} and {111}/{112} planes. Interface-generated plane surfaces (i.e., {111}/{112}) provide more thermodynamically stable and chemically active binding sites^37^,^38^ and therefore can adsorb ethanol molecules onto its abundant O^2−^ atoms better than a high-index {112} plane can (Fig. 6 and Table S2). Furthermore, a discontinuous jump in electron transfer is expected at the curvature of the stacking point of the MCS double-helix layers (Fig. 5C). The electron movement at the intricately connected MCS curvature may provide angular movement transfer (i.e., instant torque blocks) that constrains the degrees of freedom of electron transport with respect to axial movement into the outer surface of vertically oriented, continuous or non-stacked LS structures, as was evident from the diffusion coefficient value and current density of the LS- and MSC-based electrodes (Fig. 4C,D and Supplementary Section S13).

The catalytic efficiency of the developed electrodes for ethanol electrooxidation, in terms of current density, stability, surface electron movement, and molecular diffusivity, was substantially influenced by the structural characteristics of the electrode catalyst. Factors that had marked effects included a morphological architecture oriented along the longitudinal axis, the presence of multi-diffusive pores with fractal connectivity windows, and the presence of facet-dependent surface sites (i.e., high-energy exposed facets, high electron density along the nano-scale architectures, and interface planes). The effect of high-index interface planes tightly joined and closely arranged across entire mesocrystals was evident in the catalytic activities of the electrodes with CP, BC, LS, and MCS morphologies. Models of the atomic configurations and active sites of the predominant {110}, and {001} planes and their interface {110}/{001} planes were simulated by using density functional theory (Figure S20).

The geometrical structures and atomic arrangements of Co_3_O_4_ mesocrystal-based electrodes were investigated by HAADF–STEM and DFT to study the deactivation of C-NTs/Co_3_O_4_ CP and C-NTs/Co_3_O_4_ BC electrodes after multiple reuse/cycles in the EOR, as shown in Figures S19 and S21. Despite the reduction in the anodic current density after a long-term operation (>500 cycles), the catalytic active centers Co^3+^ or exposed facets were well preserved (Figure S21). The accumulation of the chemisorbed species and intermediates may gradually deactivate the electrode after >500 cycles, but the density of the catalytic sites at the top-plane insignificantly changed the catalyst {111} and {001} exposed planes (Figure S21). The HAADF–STEM micrographs also showed that the Co_3_O_4_ morphologies of the reused electrodes did not change, thus suggesting the retention of the nanostructure architectures, crystal plane surfaces, and longitudinal orientation of Co_3_O_4_ catalysts.

The density of catalytically active Co^3+^ sites arranged normal to the mesocrystal planes and the top-on-plane slapping the catalyst surfaces are key parameters of surface reactivity that markedly influenced the catalytic performance of the developed electrodes. Co^3+^ density in the catalyst planes decreased in the order {112}/{111} > {110} ≥ {001} > {110}/{001} ≥ {112} > {111}. The exposed surfaces of the {112}/{111} interface plane had more unsaturated bonds (dangling bonds) and higher surface-stabilization energies than did the other facets and interface facets. This finding is consistent with the order of catalytic reactivity of the examined morphologies (i.e., CP > LS > MCS > BC). Ethanol molecules were adsorbed onto the surfaces perpendicularly to O^2−^ atoms close to active Co^3+^ sites (Supplementary Section S20). Similarly, adsorption energy increased in the same order as the increase in Co^3+^ density of the crystal plane surfaces.

The high-index {112}/{111} interface planes in the 50-facet polyhedral catalysts had numerous exposed C/Co_3_O_4_ CP surfaces enriched with Co^3+^ active sites, which were important for effective adsorption, molecular excretion, and electron transport (Supplementary Table S2). Reactive facets containing a large number of Co^3+^ sites (i.e., the {112} side of the {112}/{111} plane) formed stronger bonds with ethanol molecules at smaller bond distances. Hence, ethanol adsorption was dependent on the active sites in the active adsorption plane (Supplementary Figure S20) in the exposed interface surfaces of the {112}/{111} and {110}/{001} planes (interface-site-dependent adsorption energy). The negative adsorption energy indicates that ethanol adsorption onto the high- and low-index crystal surface planes is an exothermic and energetically preferential behavior in an effective ethanol oxidation reaction. The change in the adsorption energy may be due to the change in atomic charges of the active adsorption surface sites of the crystal {110}, {001}, {111}, and {112} planes and their interfaces (Supplementary Table S2).

Next we examined electrostatic potential maps by using density functional theory, which showed the gradients in the surfaces in terms of the charge density of Co^2+^, Co^3+^, and O^2−^ atoms on the isosurface and the unsaturated coordination bonds (i.e., dangling bonds) at the Co^3+^ sites of the exposed {110}, {001}, {111}, and {112} planes and interfaces (Fig. 6). The change in electrostatic potential distribution on the low- and high-index planes was used to highlight the active sites; the largest deviations in electrostatic potential were found at the interfacial active centers because of the generation of oxygen vacancies in that location. Electrostatic potential mapping of the exposed plane surfaces strongly suggested that the surface oxygen atoms near O^2−^ vacancy sites had greater negative charges (an electron-rich surface) than those further from the O^2−^ vacancy sites.

The electrostatic potential map of Co^3+^ atoms showed a more pronounced charge density compared with that of the Co^2+^ atoms, indicating that Co^3+^ atoms, particularly the Co^3+^ atoms of the outer surface near the O^2−^ vacancy sites, are the most electroactive sites (Fig. 6). Therefore, more electron-rich O^2−^ vacancy sites were generated at the interface facets. As a result, interfacial active centers exhibit high electron density and large-scale electron motion across the surface atoms. The hierarchy in the surface configurations of the C/Co_3_O_4_ CP electrode oriented longitudinally could feasibly create a markedly complex {111}/{112} plane with many Co^3+^ atoms and more electron-rich O^2−^ vacancy sites in both the main- and top-layer crystal surfaces. A high adsorption energy and surface-stabilization energy, as well as an abundance of unsaturated bonds, may be achieved in the reaction, thereby producing more efficient, elastic electron transport and molecule-to-surface diffusion along these interface planes.

In summary, to achieve the practical and efficient use of direct ethanol fuel cells, this study is the first attempt to understand the role of electrocatalytic Co_3_O_4_ electrode fabrics. Atomic-scale surface architectures that were continuously ribbed and had grooves oriented along the longitudinal axis were found to improve the efficiency of ethanol electrooxidation. A new approach for the fabrication of multifunctional electrodes was demonstrated to be feasible for application in fuel cells, lithium-ion batteries, and solar cells.

## Experimental Section

### Longitudinal hierarchical engineering of catalytic Co_3_O_4_ electrodes

A simple, one-pot, hydrothermal method for synthesizing hierarchical Co_3_O_4_ nanostructure/carbon/substrate electrodes was achieved by using the hydrothermal-assisted ink method. In this method, 3D PNi (1 cm × 1 cm) and carbon glass were used as conductive scaffolds on which we directly grew C-NT or g-C/Co_3_O_4_ mesocrystal composites.

### Fabrication of C-NT/Co_3_O_4_ CP or BC/3D PNi electrodes

BC electrodes were produced from a solution of 1.5 g CoCl_2_·6H_2_O, 0.5 g urea (CO(NH_2_)_2_), and 60 mL H_2_O. CP electrodes were produced from a solution of 1.43 g CoCl_2_·6H_2_O, 3.6 g urea, and 60 mL H_2_O. C-NTs (80 mg) were added to the solutions, which were stirred for 3 h and then deposited onto 1-cm^2^ 3D PNi in a 100 mL Teflon-lined stainless-steel autoclave. The mixture was thermally treated for 12 h at 150 °C and then allowed to cool naturally to 25 °C. The product that had grown on the 3D PNi, C/Co(OH)_x_(CO_3_)_0.5_·0.11H_2_O, was washed sequentially with absolute ethanol and deionized water and then dried overnight at 60 °C. The as-prepared C/Co(OH)_x_(CO_3_)_0.5_·0.11H_2_O/CP or BC electrodes were then calcined at 400 °C with a ramp rate of 5 °C/min for 4 h under a N_2_ gas flow.

### Fabrication of C-NT/Co_3_O_4_MCS/3D PNi and g-C/Co_3_O_4_LS/3D PNi electrodes

The addition of C-NTs or g-C conductive substrates to the composition was essential for achieving the MCS and LS architectures. A mixture of 1.43 g CoCl_2_·6H_2_O, 2.94 g hexamethylenetetramine (C_6_H_12_N_4_), and 60 mL H_2_O was stirred for 10 min. Afterward, 80 mg of C-NTs or g-C was added to the solution, and the mixture was stirred for 3 h and then deposited onto 1-cm^2^ 3D PNi or in a 100 mL Teflon-lined stainless-steel autoclave. The mixture was thermally treated for 12 h at 150 °C and then allowed to cool naturally to 25 °C. The product that had grown on the 3D PNi, C-NT or g-C/Co(OH)_x_(CO_3_)_0.5_∙0.11H_2_O, was washed sequentially with absolute ethanol and deionized water and dried overnight at 60 °C. The as-prepared C/Co(OH)_x_(CO_3_)_0.5_∙0.11H_2_O/MCS and g-C/Co(OH)_x_(CO_3_)_0.5_.0.11H_2_O/LS electrodes were then calcined at 400 °C with a ramp rate of 5 °C/min for 4 h under a N_2_ gas flow.

## Additional Information

**How to cite this article**: Hassen, D. *et al.* Longitudinal Hierarchy Co_3_O_4_ Mesocrystals with High-dense Exposure Facets and Anisotropic Interfaces for Direct-Ethanol Fuel Cells. *Sci. Rep.* **6**, 24330; doi: 10.1038/srep24330 (2016).

## Supplementary Material

Supplementary Information
