# Supplementary material for: Longitudinal Hierarchy Co3O4 Mesocrystals with High-dense Exposure Facets and Anisotropic Interfaces for Direct-Ethanol Fuel Cells
Source: Sci Rep. 2016 Apr 14;6:24330. doi: 10.1038/srep24330 (PMC4830963; doi:10.1038/srep24330)
Supplement: Supplementary Information [file srep24330-s1.pdf]

Supplementary

Longitudinal Hierarchy Co_3O_4 Mesocrystals with High-dense Exposure Facets and Anisotropic Interfaces for Direct-Ethanol Fuel Cells

Diab Hassen,[1] Sherif A. El-Safty,[1,2*] Koichi Tsuchiya,[1] Abhijit Chatterjee,[3] Ahmed Elmarakbi,[4] Md A. Shenashen,[1] Masaru Sakai[3]

[1]National Institute for Materials Science (NIMS), Research Center for Strategic Materials,1-2-1 Sengen, Tsukuba-shi, Ibaraki-ken 305-0047, Japan.
[2]Graduate School of Advanced Science and Engineering, Waseda University, 3-4-1 Okubo, Shinjuku-Ku, Tokyo,169-8555, Japan

[3]Dassault System Biovia K.K., Materials Science Enterprise, ThinkPark Tower, 2-1-1 Osaki, Shinagawa-ku, Tokyo 141-6020, Japan

[4]Department of Computing, Engineering and Technology, University of Sunderland, Edinburgh Building, Chester Road, Sunderland SR1 3SD, United Kingdom

[5]Center for Research in Isotopes and Environmental Dynamics, Tsukuba University, 1-1-1 Tennodai, Tsukuba, Ibaraki 305-8572, JAPAN

TeL: +81-29-859-2135

FAX: +81-29-859-2501

E-mail: sherif.elsafty@nims.go.jp; sherif@aoni.waseda.jp

http://www.nims.go.jp/waseda/en/labo.html

http://www.nano.waseda.ac.jp/

1. Experimental Section

1.1 Materials

All investigated chemicals and materials were of analytical grade and used without further purification. Cobalt chloride hexahydrate ($CoCl_2·6H_2O$, 99%), graphite powder (98%), urea ($CO(NH_2)_2$, 99%), HMT ($C_6H_{12}N_4$), and sodium hydroxide (NaOH, 98%) were supplied by Wako Co., Ltd., Osaka, Japan. Cobalt nitrate hexahydrate ($Co(NO_3)_2·6H_2O$, 98%), ammonium nitrate (NH_4NO_3, 99%), absolute ethanol (C_2H_5OH, 99.5%), nitric acid (HNO_3, 61%), sulfuric acid (H_2SO_4, 95%), hydrochloric acid (HCl, 36%), and hydrogen peroxide (H_2O_2, 30%) were purchased from Nacalai Tesque Co., Japan. The MWCNTs (98%) were obtained from Sigma-Aldrich Company Ltd., USA, whereas the potassium permanganate ($KMnO_4$, 99.5%) was purchased from Tokyo Chemical Industry Company (TCI), Ltd., Japan. The 3D porous Ni foam (3D PNi) (1 cm × 1 cm) was produced by TCI as a conductive scaffold on which the nanocomposites were grown. Prior to synthesis, the 3D PNi substrates were repeatedly rinsed by sonication in concentrated HCl solution, ethanol, and Milli-Q water in succession and then dried for 12 h at 60 °C.

1.2. Purification and functionalization of MWCNTs

The raw MWCNTs were initially mixed with HNO_3/H_2SO_4 solutions at a 1:3 ratio. The mixture was then ultrasonicated at 50 °C for 5 h and then refluxed at 110 °C for 3 h. Afterward, the resulting mixture was diluted at pH 7 using deionized water. The solid (i.e., oxidized MWCNTs) was collected by centrifugation and dried at 65 °C overnight for later use.

1.3. Preparation of graphene oxide

Graphene oxide was successfully synthesized from graphite powder in accordance with the slightly modified Hummers method[1]. Typically, 2 g of graphite powder (~40 µm) was reacted with a strong oxidizing solution of concentrated H_2SO_4 and HNO_3 (100 mL, 1:1 v/v) by vigorous stirring for 1 h at 25 °C. Afterward, the solution was placed in an ice-water bath, and 7 g of $KMnO_4$ was slowly added to the solution under stirring for 2 h. The mixture was then ultrasonicated for 6 h at 40 °C to avoid obtaining a homogeneous reaction solution. Deionized water (350 mL) was then mixed with the formed gel, and the whole mixture was stirred at 80 °C for 1 h. Subsequently, 120 mL of 30% H_2O_2 and 50 mL of 15% HCl solutions were added to the mixture and then stirred for 10 min. The gel mixture was allowed to stand until brownish precipitation. The solid materials were washed repeatedly with double-distilled water and dried overnight at 60 °C prior to the next investigation.

1.4. Preparation of C-NT/CoO CPs/3D PNi nanostructures

A simple, one-pot method for the synthesis of C-NT/CoO CPs/3D PNi electrode was achieved through a microwave-assisted technique. In this method, 0.1 M cobalt nitrate hexahydrate and 0.35 M HMT were mixed with 37 mL of deionized water while stirring for 10 min. Then, 80 mg of oxidized C-NTs were immersed into the above solution and vigorously stirred for 3 h. The mixture was subsequently transferred into a Teflon-lined autoclave containing a 3D PNi sheet (Scheme 1) and then subjected to microwave irradiation (600 W) at 160 °C for 1 h. The collected sample was carefully washed with ethanol and deionized water, dried at 60 °C overnight, and finally calcined at 400 °C for 4 h in air.

1.5. Fabrication of C-NT or g-C/Co_3O_4 or CoO/GC

To investigate the effect of the carrier substrate on EOR efficiency using a longitudinal electrode design, several electrochemical experiments were conducted using C-NT or g-C/Co_3O_4 or CoO/GC electrodes at electrochemical conditions similar to those applied to the C-NT or g-C/Co_3O_4 or CoO/3D PNi electrode assays. The thin-film-layered C-NT or g-C/Co_3O_4 or CoO/GC electrode was fabricated by dispersing the active catalyst powder onto a GC substrate through a heterogeneous-assisted ink-deposition method at 25 °C. The homogeneous catalyst ink was prepared by mixing 5 mg of active catalyst to 50 µL of 5 wt% Nafion solution employed as a binder in 2 mL of Milli-Q water under ultrasonication for at least 30 min. The catalyst ink (4 µL) was then loaded onto the active area of a GC (Φ = 3 mm) at ~0.143 mg/cm^2. The catalyst ink-loaded GC electrode was dried in a sealed oven at 50 °C to allow the formation of uniform catalyst layers over the GC substrate area.

2. Characterization

The morphologies of the annealed samples were investigated by FE-SEM (JEOL Model 6500) at 15 kV. The C-NT or g-C/Co_3O_4 or CoO/3D PNi electrodes were fixed onto the FE-SEM stage using carbon tape before insertion into the FE-SEM chamber. The ion sputter (Hitachi E-1030) was used to deposit thin-layered Pt films on electrodes at 25 °C.

A focused ion beam (FIB) system (JEM-9320FIB) operated at accelerating voltages from 5–30 kV with variable steps of 5 kV and magnification ranging from 150× to 300000×. The orientation axis (X and Y) of the powder samples containing C-NT or g-C/Co_3O_4 or CoO catalysts can be changed within ±1.2 mm through a tilt angle of ± 60°. The samples were inserted inside the FIB machine using a bulk-sample holder (8 × 8 mm^2) after deposition by a carbon protection layer. Before FIB investigation, the powder samples of the C-NT or g-C/Co_3O_4 or CoO catalysts were mixed with small amounts of epoxy (Gatan, Inc.) onto a small silicon wafer using a fine eyelash probe to form very

thin films on the silicon substrate (Figures 2 and S7). Each thin film was baked on a hot plate at 130 °C for 10 min and subsequently coated with a uniformly thin carbon layer of about 30 nm. The samples were inserted into the FIB microscope operated at 30 kV and then roughly milled on both sides until a final thickness of 2 μm using −1.5° and +1.5° tilts. Afterward, the C-NT or g-C/Co_3O_4 or CoO sample was cut and removed from the FIB system for subsequent HAADF–STEM microscopy.

HAADF-STEM was employed to perform (i) TEM and (ii) STEM, (iii) EDS for elemental mapping, and (iv) electron diffraction (ED). The HAADF–STEM micrographs were recorded using a JEM-ARM200F-G instrument supplied with aberration correctors at the illumination and imaging lens systems to observe TEM/STEM images at high resolution. The HAADF–STEM microscope was also equipped with a monochromated electron gun and supported by electron energy-loss spectroscopy at a high-energy resolution. Specifically, the cross-section specimens for HAADF–STEM was prepared by FIB system milling. The fine trapped probes typically sharpened the sample in the parallel direction of the longitudinal *c*-axis. The well-prepared FIB samples were attached to a silver grid by epoxy materials using a pick-up system. The C-NT or g-C/Co_3O_4 or CoO attached to the silver grid was inserted again into the FIB system to produce a 100 nm-thick layer. The sample was thinned from both sides by using alternate beams with variable intensity until the final thickness of 100 nm. The 100 nm sample was viewed under the HAADF–STEM microscope to record the cross-sectional images.

The surface properties of the material involving the pore structure distribution and surface area were estimated by N_2 adsorption–desorption isotherms at 77 K using a BELSORP36 analyzer (JP. BEL Co., Ltd.). The samples were thermally treated at 200 °C for at least 6 h under N_2 atmosphere. The specific surface area (S_{BET}) was calculated using the Brunauer–Emmett–Teller (BET) method with multipoint adsorption data from the linear section of the N_2 adsorption isotherm. The pore size distribution was determined using nonlocal DFT (NLDFT).

The structural geometry of the catalysts was further examined by WA-XRD. The WA-XRD patterns were recorded using a 18 kW diffractometer (Bruker D8 Advance) at scan rate of 10°/min with monochromated $Cu_{K\alpha}$-X-radiation (λ = 1.54178 Å). The DIFRAC plus Evaluation Package (EVA) software with the PDF-2 Release 2009 databases provided by Bruker AXS was used to analyze the diffraction and structure analysis diffraction data. The TOPAS package program was applied to integrate various types of X-ray diffraction (XRD) analyses.

XPS analysis was conducted on a PHI Quantera SXM (ULVAC-PHI) instrument (Perkin–Elmer Co., USA) equipped with Al Kα as an X-ray source for excitation (1.5 mm × 0.1 mm, 15 kV, 50 W)

under a pressure of 4×10^{-8} Pa. A thin film of the sample was deposited on a Si slide before the start of analysis.

Raman spectroscopy (HR Micro Raman spectrometer, Horiba, Jobin Yvon) was conducted using an Ar ion laser at 633 nm. A CCD (charge coupled device) camera detection system and the LabSpec-3.01C software package were used for data acquisition and analysis, respectively. To ensure the accuracy and precision of the Raman spectra, 10 scans of 5 s from 300 cm^{-1} to 1,600 cm^{-1} were recorded.

TG and DTA were achieved using a simultaneous DTA–TG Apparatus TG-60 (Shimadzu, Japan).

3. Electrochemical measurements

Electrochemical measurements were obtained in a home-made electrochemical cell using mercury/mercury oxide (Hg/HgO, 1 M NaOH) and platinum wire (Φ = 0.1 mm) as the reference and counter electrodes, respectively. The active catalyst C-NT or g-C/Co_3O_4 or CoO grown on 3D PNi with surface structures of CPs, LSs, MCSs, and BCs and with active loading of l.5 mg served as the working electrodes for electrochemical investigation. The data were recorded using a Zennium/ZAHNER electrochemical work station (Elektrik GmbH & Co. KG) controlled by the Thales Z 2.0 software. Initially, all of the working electrodes were cycled at least 10 times at a scan rate of 50 mV s^{-1} until the signals were stabilized. Then, the CV data were collected. Current density refers to the geometrical surface area of the investigated working electrodes (1 cm^2). The measured potentials were reported with respect to the Hg/HgO reference electrode. The freshly prepared electrolyte (0.5 M NaOH) was de-aerated by bubbling a slow stream of purified N_2 above the electrolyte in the electrochemical glass cell. The N_2 flow was maintained during the electrochemical measurements to ensure an N_2-saturated electrolyte. To guarantee the reproducibility of the recorded results, freshly prepared electrolyte solutions were used for every electrochemical measurement. Chronoamperometry tests (CA) were conducted to evaluate electrode stability during the ethanol electrooxidation reaction. CA measurements were obtained by applying a constant potential of 0.7 V versus Hg/HgO in the presence of 0.5 M ethanol. EIS measurements were performed at a frequency range of 100 kHz–0.01 Hz with a 5 mV amplitude and an open circuit potential.

4. Mathematical modelling

DFT is a promising approach to effectively illustrate the electronic correlation effects. In this study, all calculations investigated by DFT were performed in accordance with the DMol3 of BIOVIA Dassault systems[2,3]. The exchange-correlation energy function was represented by the Perdew–Burke–Ernzerhof (PBE) formalism[4]. The Kohn–Sham equation was expanded in a double numeric

quality basis set (DNP) with polarization functions. To consider the relativistic effect, the DFT Semi-core Pseudo-potentials[5] were used for the treatment of the core electrons of the doped clusters. The orbital cutoff range and Fermi smearing were selected as 5.0 Å and 0.001 Ha, respectively. The self-consistent-field (SCF) procedures were performed to obtain well-converged geometrical and electronic structures at a convergence criterion of 10^{-6} a.u. The energy, maximum force, and maximum displacement convergence were set to 10^{-6} Ha, 0.002 Ha/Å, and 0.005 Å, respectively. Meanwhile, the electrostatic site potential is a measure of the Coulomb interaction per unit charge experienced by an ion at a given position in space. DFT was also used to calculate the electrostatic potential (EP) distribution. Modeling was performed to show a physical quantitative survey at each point on the isosurfaces using a feature of the surface-charging map. Typically, the isosurfaces of the electron densities were colored on the basis of EP intensities (EPI) using a lattice representation in which the charges are mapped on the cubic lattice in the so called contour where the EP is calculated. The slab model was constructed with nine atomic layers of each catalyst (Figure 6). To compare the active center within the structure, oxygen atoms at the surface and subsurface layers were involved in the stoichiometric mode. EP was investigated over the range of −0.06 eV to +0.6 eV as shown in the optimized model

5. Hydrothermal-assisted formation of hierarchal Co_3O_4 nanocomposites

Scheme 1 shows the morphological evolution of the designed electrodes. The C-NT or g-C/Co_3O_4 nanohybrid structures derived from the hierarchical metal framework grown directly on the 3D PNi substrate with robust mechanical adhesion were obtained after adequate pyrolysis of the as-synthesized C-NT or g-C/ $Co(OH)_x(CO_3)_{0.5}·0.11H_2O$/3D PNi electrodes at 400 °C for 4 h (additional details are found in the Experimental section). We adopted scalable and flexible methods to fabricate morphology-controlled nanohybrids along the longitudinal direction vertically oriented toward the 3D PNi skeleton. In these processes, the surface morphologies of the CPs and BCs in the NR architecture and MCSs and LSs in nanosheet dominates were engineered. GO sheets (g-C) or functionalized multi-walled carbon nanotube (C-NT) counterparts acted as carbon supports. Basically, the unique structures of the fabricated electrodes were contributed by the directing basic salt (urea and HMT) and cobalt precursor. To well understand our unique and reasonable electrode building along the longitudinal scales, Table S1 summarizes some of the reported materials based sheets, nanowires, and rod structures by different routes.

Under hydrothermal treatment conditions, fabrication of self-supported (i.e., C-NT or g-C/Co_3O_4 or CoO/substrate) electrodes, Co_3O_4 or CoO and C-NT or g-C/Co_3O_4 or CoO hybrids could be

hierarchically controlled to yield morphological features similar to those of BC-NR, CP-NR, LS, and MCS structures with uniformly spaced inner pores of micro- and mesoscale sizes (0.5–65.8 nm), as evidenced from field emission scanning electron microscopy (FE-SEM) micrographs (Figures S1-S4), thermogravimetry (TG)–differential thermal analysis (DTA), wide-angle powder X-ray diffraction (WA-XRD), and N_2 isotherms; see Figures S5-S7). To investigate the atomic core-level organization of the geometrical C/Co_3O_4 mesocrystals hybrids in terms of chemical bonding, structural environment, and elemental state, we conducted X-ray photoelectron spectroscopy (XPS) analysis and Raman spectroscopy of the C/Co_3O_4 CPs (Figure S8).

The FE-SEM and HAADF–STEM micrographs (Figures 1–2, S1–S4, and S9–S10) indicate that the reaction time as well as the growth temperature is quite sufficient for the immobilization and nucleation of the free standing nanostructures. In addition, the metal particles were bonded to the surface group of the counterparts by electrostatic interactions during the hydrothermal treatment (Schemes 1A-a, B-a, and B-b). These oxygen functional groups provide thorough dispersion of the counterparts into the reaction solution and offer reliable mediators for the successful growth of Co_3O_4 mesocrystals on the counterpart scaffolds. The porosity of the formed nanostructure was greatly enhanced after decomposition at relatively high temperatures from the release of gases, adsorbed water, and organic molecules. This result indicates enhanced utilization of the active material. Hence, remarkable improvement in their electrochemical performance was expected. Moreover, such a unique structure provides well-defined pathways for the electrolyte solution to pass through the graphene layer and along the carbon tubes, leading to fast electron transport. Interestingly, the controlled longitudinal growth of the C-NT or g-C/Co_3O_4 or CoO/3D PNi electrodes generates more favorably exposed surfaces and interfaces containing an extensive domain of Co^{3+} active sites. This effect enhances the kinetics of the EOR, as evidenced by the HR-HAAF-STEM micrographs (Figures S10).

Table S1 Comparison showing the reported synthesis routes for the fabrication of Co_3O_4 like sheets, nanowires, and rod structures

Active materials	Precursors	Synthesis conditions	Product shape	Reference
Co_3O_4	$Co(NO_3)_2.6H_2O$ and HMT	Hydrothermal treatment at 120 °C for 12 h	Nanosheet arrays	6
Co_3O_4 or Co_3O_4/C	$Co(NO_3)_2$. $6H_2O$, NH_4F and urea	120 ^oC for 9 h	Nanoneedle arrays	7
$NiCo_2O_4$@ RGO	$NiCl_2.6H_2O$, $CoCl_2.6H_2O$, the pH was adjusted to 11 using 25% NH_4OH	Hydrothermal treatment at 180 °C to 24 h	Monolayer	8
Co_3O_4@C	$CoCl_2{\cdot}6H_2O$, carbon nanosheets and urea,	Hydrothermal treatment at 120 °C for 6 h	nanosheets	9
Co_3O_4@ graphene	$Co(NO_3)_2.6H_2O$ and HMT	Microwave irradiation at 180 ^oC for 5 min	Nanosheets	10
Co_3O_4	$Co(NO_3)_2 \cdot 6H_2O$, NH_4F and urea	Hydrothermal, 120 ^oC for 9 h,	Nanoarrays	11
$NiCo_2O_4$	Ni $(NO_3)_2{\cdot}6H_2O$, $Co(NO_3)_2{\cdot}6H_2O$, and urea	Hydrothermal treatment at 120 °C for 6 h	Nanoneedle arrays	12
Co_3O_4	$Co(NO_3)_2.6H_2O$, urea, and CTAB	Microwave oven irradiation for 20 min	Nanorod	13
Co_3O_4	$Co(NO_3)_2$, NH_4F and $CO(NH_2)_2$	Hydrothermal treatment at 120 °C for 9 h	Nanowire array	14
Co_3O_4/C	Cobalt(II) acetate tetrahydrate and dipotassium 2,6-naphthalene-dicarboxylate	Hydrothermal treatment at 80 °C for 20 h	Nanowire array	15
CoO	$Co(NO_3)_2 \cdot 6H_2O$ and CO(NH2)2	Hydrothermal treatment at 365 K for 8 h	Nanowire Arrays	16
Co_3O_4	$Co(NO_3)_2.6H_2O$, Triton X-100, and urea	Hydrothermal treatment at 120 ^oC for 24 h	Ultralayered	17
$NiCo_2O_4$	$Ni(NO_3)_2{\cdot}6H_2O$, $Co(NO_3)_2{\cdot}6H_2O$ and HMT.	Hydrothermal treatment at 90 ^oC for 10 h	Nanosheet	18
R-$Co(OH)_2$	$Co(NO_3)_2{\cdot}6H_2O$, NH_4F , $CO(NH_2)_2$	Hydrothermal, 120 ^oC for 6 h	Nanowire Arrays	19
$NixCo_{3\text{-}x}O_4$	$Co(NO_3)_2$. $6H_2O$, $Ni(NO_3)_2.6H_2O$, and NH_4NO_3	Hydrothermal treatment for 12 at 90 ^oC.	Nanowire Arrays	20

Supplementary S1

Addition of urea and hexamethylenetetramine (HMT, $C_6H_{12}N_4$) agents is essential to achieve NR-, LS- and MCS-like morphologies, determine the orientation of growth of Co_3O_4 or CoO mesocrystals, and construct the atomic structure along with the active exposure of low- and high-index single and interface plane surfaces (Figures S1-S4). The dose of urea species significantly affects the formation of CP- (high urea concentration) and BC- (low urea concentration) NR Co_3O_4 structures with specific crystal planes and, consequently, the catalytic performance in EOR. In addition, the proposed growth mechanism of Co_3O_4 or CoO mesocrystals in hybrid or electrode fabrics most likely involves two stages: (i) homogeneous nucleation of cobalt seeds potentially forming thermodynamically stable and actively centered nuclei sites of $Co(CO_3)_{0.5}(OH)_x{\cdot}0.11H_2O$ composition domains and (ii) time- and temperature-dependent controlled growth of active and stable-centered seeds to achieve longitudinal growth around the c-axes in the final structures.

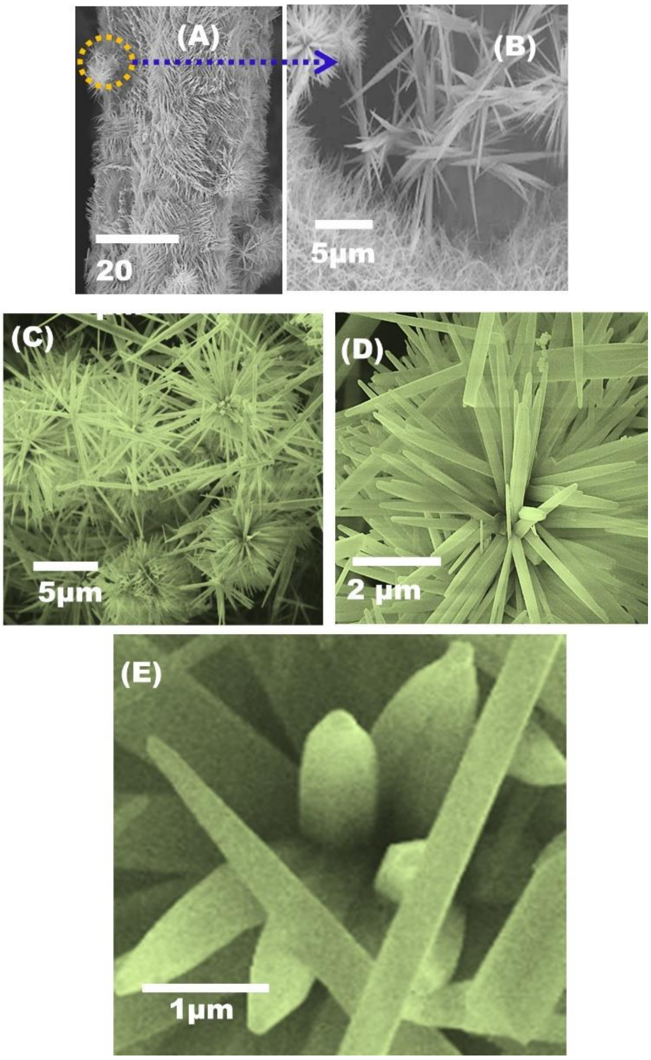

Fig. S1. (A–E) Low- and high-magnification top-view SEM images of the hierarchically controlled pristine Co_3O_4 nanostructures aligned vertically onto 3D PNi foam (A &B) along the longitudinal axis. (C-E) SEM micrographs of the Co_3O_4 BCs resembling giant bamboo trees throughout the NR columns. (E) The sharp-edged Co_3O_4 BCs architecture along the longitudinal scales.

Supplementary S2

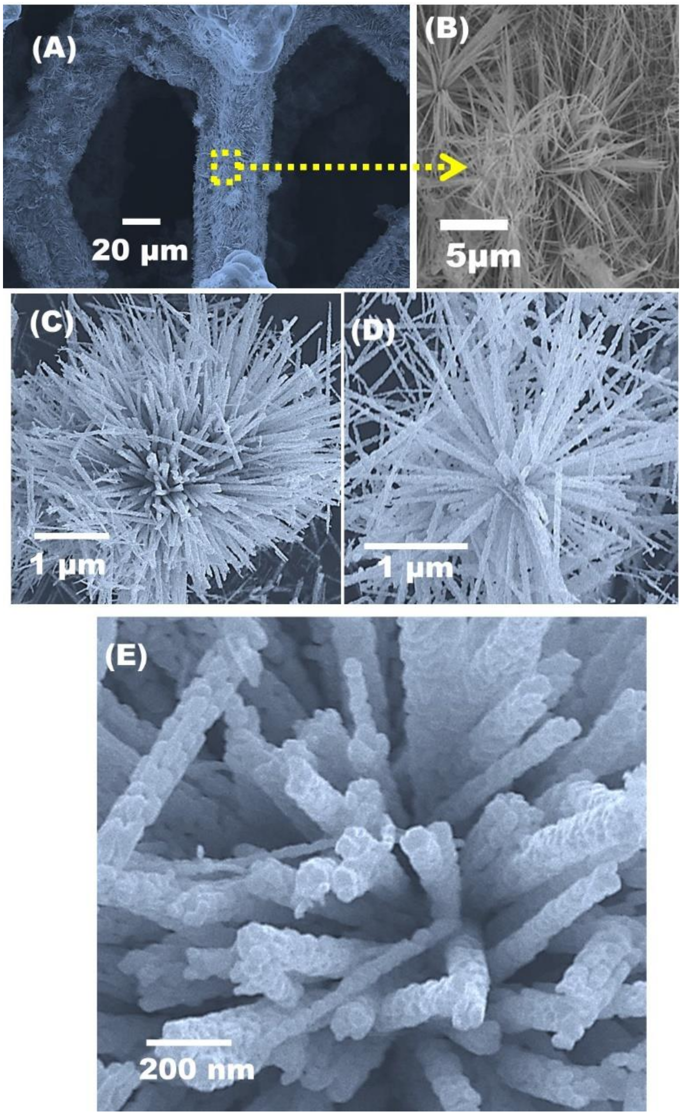

Fig. S2. (A–E) shows a dense aggregation of particle unit blocks of Co_3O_4 or CoO CPs mesocrystals aligned vertically onto 3D PNi foam (A &B) along the longitudinal axis. This aggregation generates a unique molecular carbon/Co_3O_4 (C,D) or CoO (E) structure of CPs synthesized in a vertical fashion with respect to the underlying 3D PNi substrate. Significantly, addition of the C-NT counterpart supports did not affect the stable molecular level morphology of Co_3O_4 or CoO CPs, but decreased the thickness of the NR sizes (C-E).

Supplementary S3

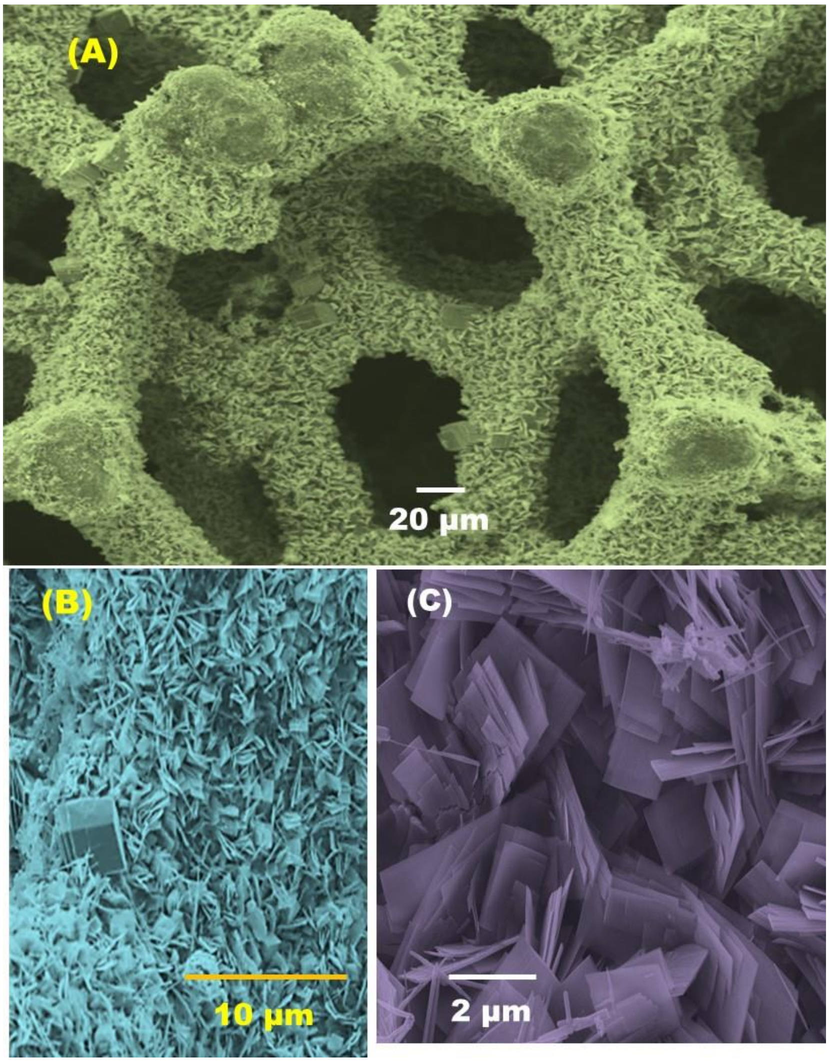

Fig. S3. (A-C) Top-view low- and high-magnification FE–SEM micrographs of the hierarchical g-C /Co_3O_4 LS/3D PNi electrodes fabricated along the longitudinal axis via the one-pot hydrothermal approach. The images illustrate the scalability of our investigated synthesis route. The images display the efficient surface control of the sheet structure along the longitudinal axis.

Supplementary S4

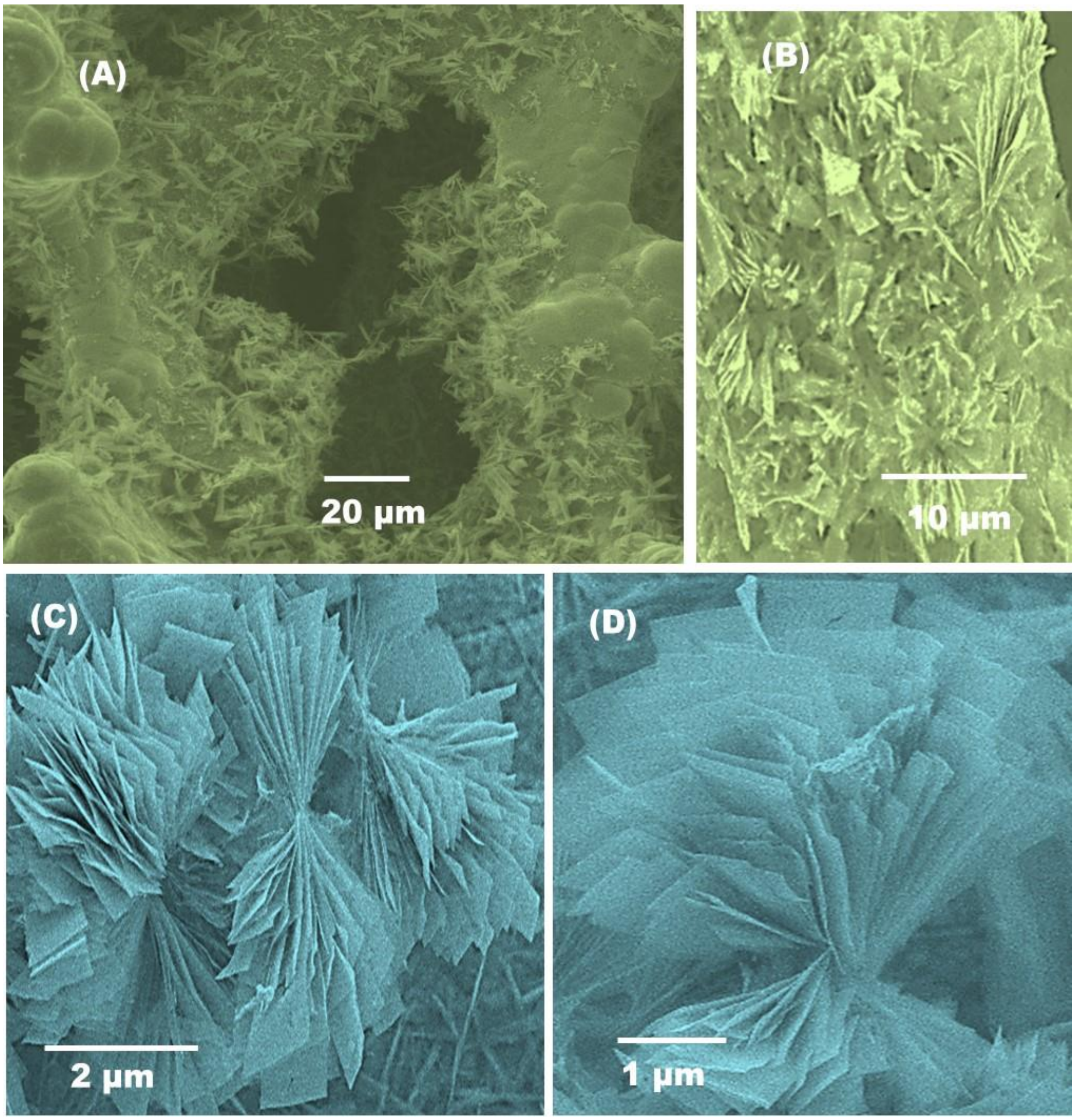

Fig. S4. (A-D) Top-view low- and high-magnification FE–SEM micrographs of the hierarchical g-C /Co_3O_4 MCSSs/3D PNi electrodes fabricated along the longitudinal axis via the one-pot hydrothermal approach. The geometric features of the hierarchical C/Co_3O_4 MCSSs arranged axially around the axial stacking center at the discrete point of surface direction along the longitudinal axis of C-NTs synthesized via one-pot hydrothermal approach.

Supplementary S5

To further illustrate the thermal conversion from the precursors $Co(OH)_x(CO_3)_{0.5}·0.11H_2O$ to the final product Co_3O_4, thermal gravimetric analysis (TGA) was conducted. Through this approach, the thermal properties and chemical composition of the $Co(OH)_x(CO_3)_{0.5}.0.11H_2O$ CP precursor powder were initially determined (Figure S5). A DSC plot of the sample shows two exothermal peaks located in the gravimetric gain region centered at 228 and 320.5 °C. At around 395 °C, the total weight loss of the sample is about 17.45%, which was ascribed to the successive release of CO_2 during the thermal decomposition of the $Co(OH)_x(CO_3)_{0.5}·0.11H_2O$ to Co_3O_4 and the evaporation of adsorbed water molecules. As revealed by thermogravimetry (TG)–differential thermal analysis (DTA), no weight loss was observed for the precursor after 400 °C, indicating complete decomposition from the precursor to Co_3O_4. On the basis of the TGA data, we performed the thermal heat treatment of the sample at 400 °C.

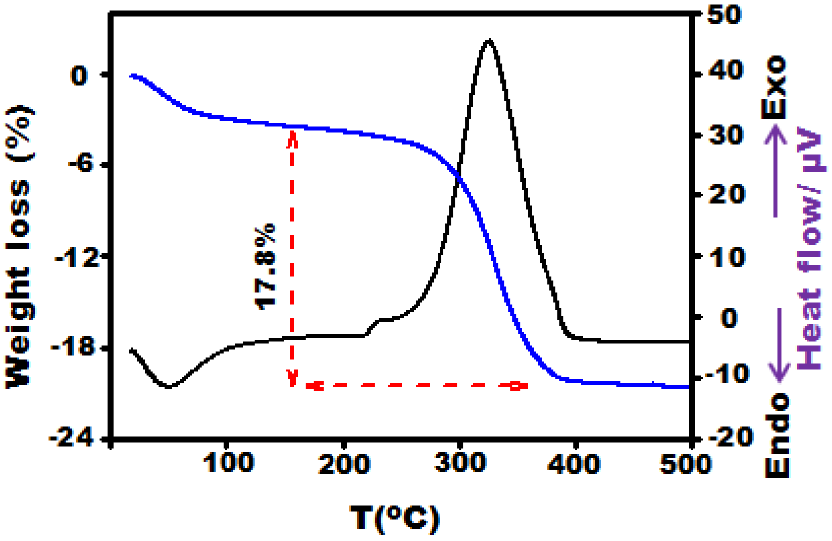

Fig. S5. TG–DTA curves measured for the $Co(OH)_x(CO_3)_{0.5}·0.11H_2O$ CP hierarchical structure revealing the thermal events that occurred. These curves indicate the total weight loss of the target sample during heat treatment.

Supplementary S6

Figures S6A and S6B show the powder XRD patterns of the as-prepared cobalt carbonate hydroxides under urea/HMT-assisted hydrothermal process and the final products obtained after annealing. Under further thermal treatment at ~400 °C (Figure S6), the coordination by H-bonding interactions of the orthorhombic cobalt basic carbonate (JCPDS No. 48-0083) mesocrystals [i.e., $Co(OH)_x(CO_3)_{0.5}.0.11H_2O$] preferably rearranged to generate Co^{2+} and Co^{3+} bound via tetrahedral and octahedral coordination to four and six oxygen ions, respectively, in the entirely single crystal face-centered-cubic [fcc, *Fd3m*, JCPDS 42-1467 and fcc, *Fm3m*, JCPDS 65-2902] Co_3O_4 and CoO structures[21,22]. These findings were noted from the wide-angle powder X-ray diffraction (WA-XRD) profiles. The WA-XRD spectra of the carbon/$Co(OH)_x(CO_3)_{0.5}\cdot 0.11H_2O$ mesocrystals show diffraction peaks at 26° and 26.5°, corresponding to the (200) reflection of C-NTs and g-C, respectively (Figure S6A- S6B). This result indicates that the single-crystal structure stabilities of both Co_3O_4 and CoO structures increase with addition of C-NT or g-C conductive substrates because of the strong electrostatic interactions between the cobalt oxide mesocrystals and the surface oxygen groups of the counterparts.

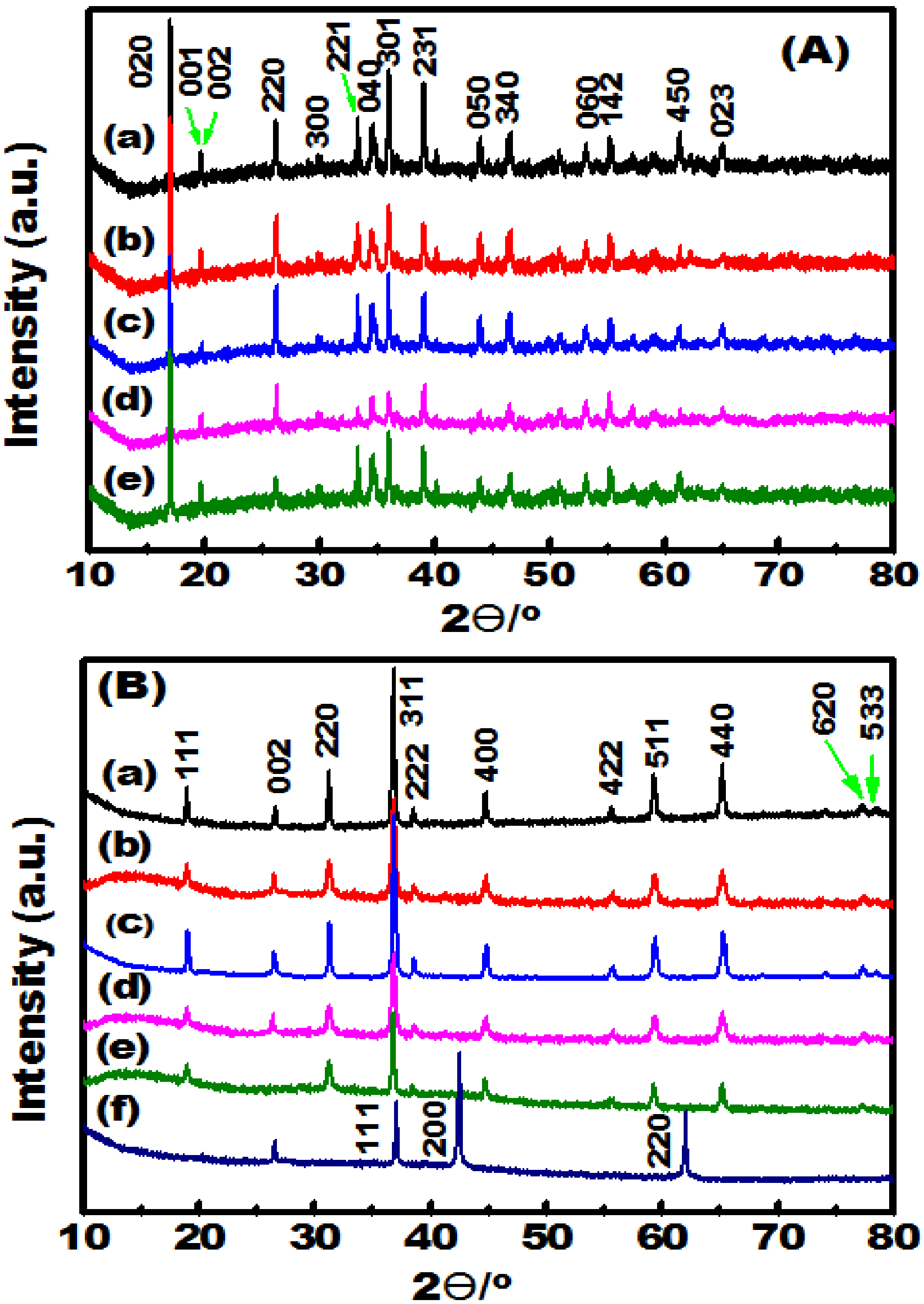

Fig. S6. (A, B) WA-XRD of the as-synthesized and calcined samples assigned according to the databases of the International Center for Diffraction Data provided by Bruker. (A) XRD patterns of $Co(OH)_x(CO_3)_{0.5}{\cdot}0.11H_2O$/carbon orthorhombic cobalt basic carbonate phase of the engineered surface structures of CPs (a), BCs (b), MCSs (c), and LSs (d). (e) Orthorhombic cobalt basic carbonate phase of the bare $Co(OH)_x(CO_3)_{0.5}{\cdot}0.11H_2O$ CPs. (B) XRD spectra of the face-centered-cubic phase of the final product *Fd3m* C-NT or g-C/Co_3O_4 and *Fm3m* C-NT/CoO of the morphological architectures of CPs (a), BCs (b), MCSs (c), and LSs (d). Spectra of bare Co_3O_4 CPs (e) and C-NT/CoO CPs (f).

Supplementary S7

N_2 adsorption/desorption isotherms obtained at 77 K are presented in Figure S6. The type IV isotherms of the calcined C-NT or g-C/Co_3O_4 or CoO powder samples that featured mesoporous materials are evident[23]. The hysteresis loop at the relative pressure range of 0.88–1.0 P/Po, indicates the association of the macroporous space voids with the morphological hierarchy. The pore-size distribution of the C-NT or g-C/Co_3O_4 or CoO samples was measured using the NLDFT theory (Insets a–e). The existence of the micropore space with C-NT or g-C/Co_3O_4 or CoO samples (Figures S7A–S6E) can be possibly due to the residual growth of cobalt oxide mesocrystals on the carbon support. Notably, the hybrids featured specific surface areas (S_{BET}) of 46.5, 34.5, 40.3, 29.1, 23.8, and 19.4 m^2g^{-1} for the C-NT/Co_3O_4 CPs, C-NT/Co_3O_4 MCSs, g-C/Co_3O_4 LSs, C-NT/Co_3O_4 BCs, C-NT/CoO CPs, and Co_3O_4 CPs, respectively. The surface area of the C/Co_3O_4 CPs is higher than that of bare Co_3O_4, suggesting the robust formation of Co_3O_4 or CoO/carbon structures with high degree of surface uniformity. In general, the N_2 isothermal results indicate that our synthesis strategy effectively controls the hierarchical porous structures with multi-functional micro-, meso-, and macro-cavity pores. The distribution of these pores across the hierarchical porosity may create a potential accessibility of ethanol molecules into the electrode matrices. Hierarchical C-NT or g-C/Co_3O_4 or CoO morphologies were developed with uniformly spaced inner pores of micro- and mesoscale sizes (0.5–65.8 nm) (N_2 isotherms; Supplementary Section S6). Insertion of C-NT or g-C counterpart supports into the Co_3O_4 or CoO frameworks leads to (i) formation of micropore sizes, (ii) double enhancement of the surface-area-to-volume ratios of exposed catalytic surfaces, and (iii) controlled uniformity of the mesopore distribution (Figures S7A–S7F). These surface structural features enable the multi-accessible windows of ethanol fluids to bind with interior active Co^{3+} sites during the EOR process.

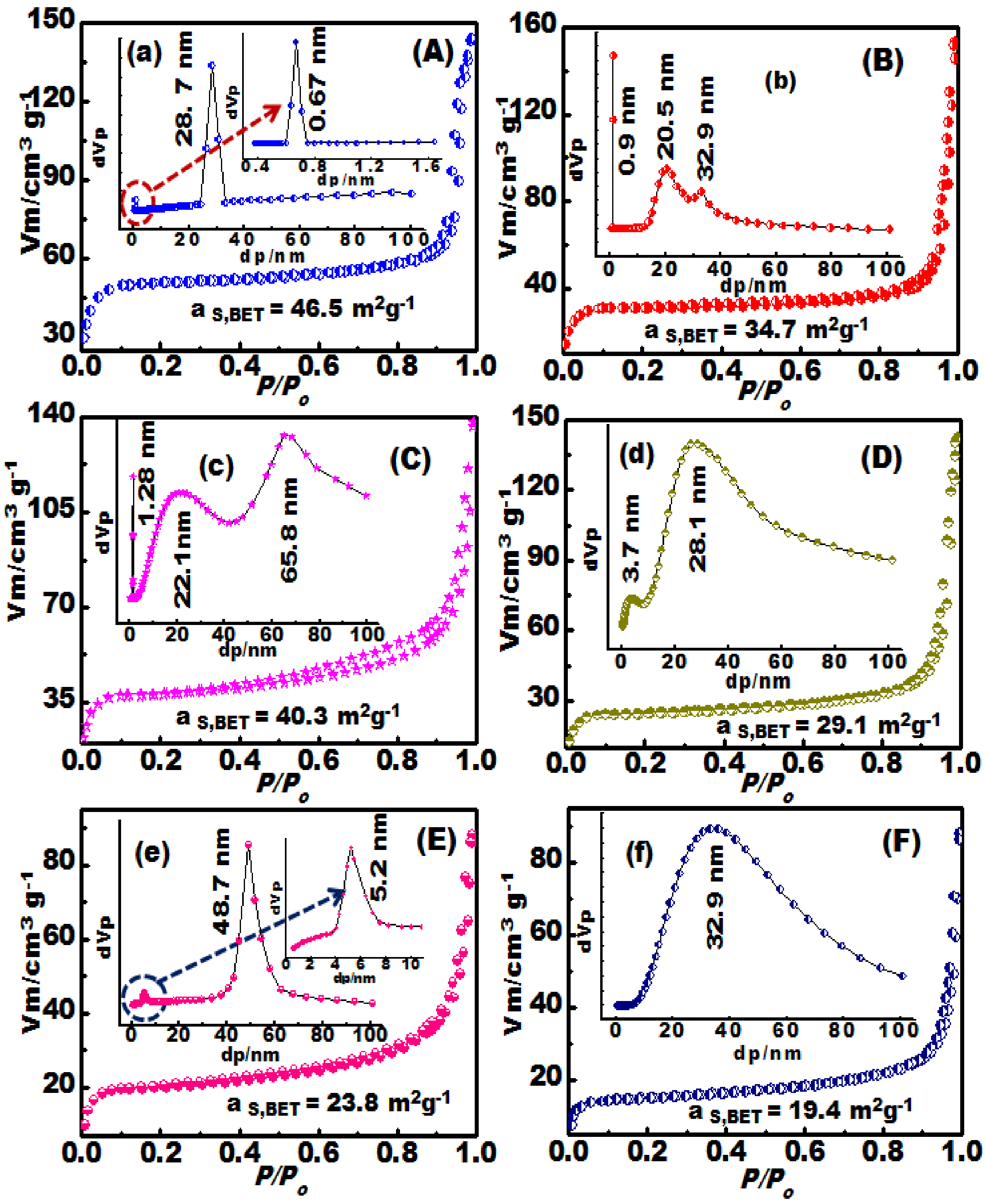

Fig. S7. (A–E) N_2-adsorption/desorption isotherms of the calcined samples showing the textural properties including the specific surface area and pore size distribution measured at 77K. (A) C-NT/Co_3O_4 CPs, (B) C-NT/Co_3O_4 MCSs, (C) g-C/Co_3O_4 LSs, (D) C-NT/Co_3O_4 BCs (E) C-NT/CoO CPs, and (F) pristine Co_3O_4 CPs. The specific surface area ($S_{,BET}$) of the samples was collected of the liner part of the related absorption loops. The inserts (a-f) represent the corresponding pore size distribution analyzed by using NLDFT theory from the N_2-adsorption/desorption hysteresis.

Supplementary S8

To investigate the atomic core-level organization of the geometrical C-NT or g-C/Co_3O_4 mesocrystal hybrids in terms of chemical bonding, structural environment, and elemental state, we conducted X-ray photoelectron spectroscopy (XPS) analysis and Raman spectroscopy of the C-NT/Co_3O_4 CPs (Figure S8). The wide-survey XPS spectrum of the Co_3O_4/C-NT shows the composition and ratios of C, Co, and O (Figure S8A). Distinctive peaks of Co 2p, Co LMM, Co 3s, and Co 3p signals are characteristic of Co_3O_4 CPs. The high-resolution XPS spectra of Co 2p exhibit two distinctive peaks centered at 795.3 and 780.3 eV, with a 15.0 eV energy difference, corresponding to the Co 2p1/2 and Co 2p3/2 peaks, respectively, in a pure Co_3O_4 crystal phase[24,25] (Figure S8B). The O1s peak (Figure S8C) could be deconvoluted into three singlet peaks centered at 528.9, 531.2, and 533.7 eV, which are assigned to the structural environment of oxygen atoms coordinated in the hydroxyl groups, absorbed water, and Co_3O_4 surface crystal, respectively[25]. Enhancement of the intensity of the O1s peak at 531.2 eV indicates the contribution of hydroxyl groups of the functional C support surfaces. The high-resolution spectra of C1s at 285.1 eV displays three peaks (Figure S8D) at 284.9, 286.6, and 287.8 eV denoting carbon in C−O, C=O, and sp^2 C−C bonds, respectively[26]. The Raman spectra of the C/Co_3O_4 CPs and C/Co_3O_4 LSs hybrids show six well-defined peaks (Figure S8E). The peaks that centered at 481.2, 520.9, 619, and 684 cm^{-1} are respectively attributed to the Eg, F_{2g}^{1}, F_{2g}^{2}, and A_{1g} vibrational modes of the Co_3O_4NC bands[27]. The three distinctive peaks of the C/CoO CPs at 464.6, 516.8, and 658.8 cm^{-1} correspond to the E_g, F_{2g}^{1}, A_{1g} vibrational modes, respectively, of the CoO bands[27]. The highly resolved and intense peaks of the C-NT or g-C/Co_3O_4 and C-NT/CoO mesocrystals at 1349.3 (D band) and 1600.1 cm^{-1} (G band) can be attributed to the E_{2g} vibrational mode of the second-order band (sp^2) and crystal defects of the bonded graphitic carbon, respectively [28,29].

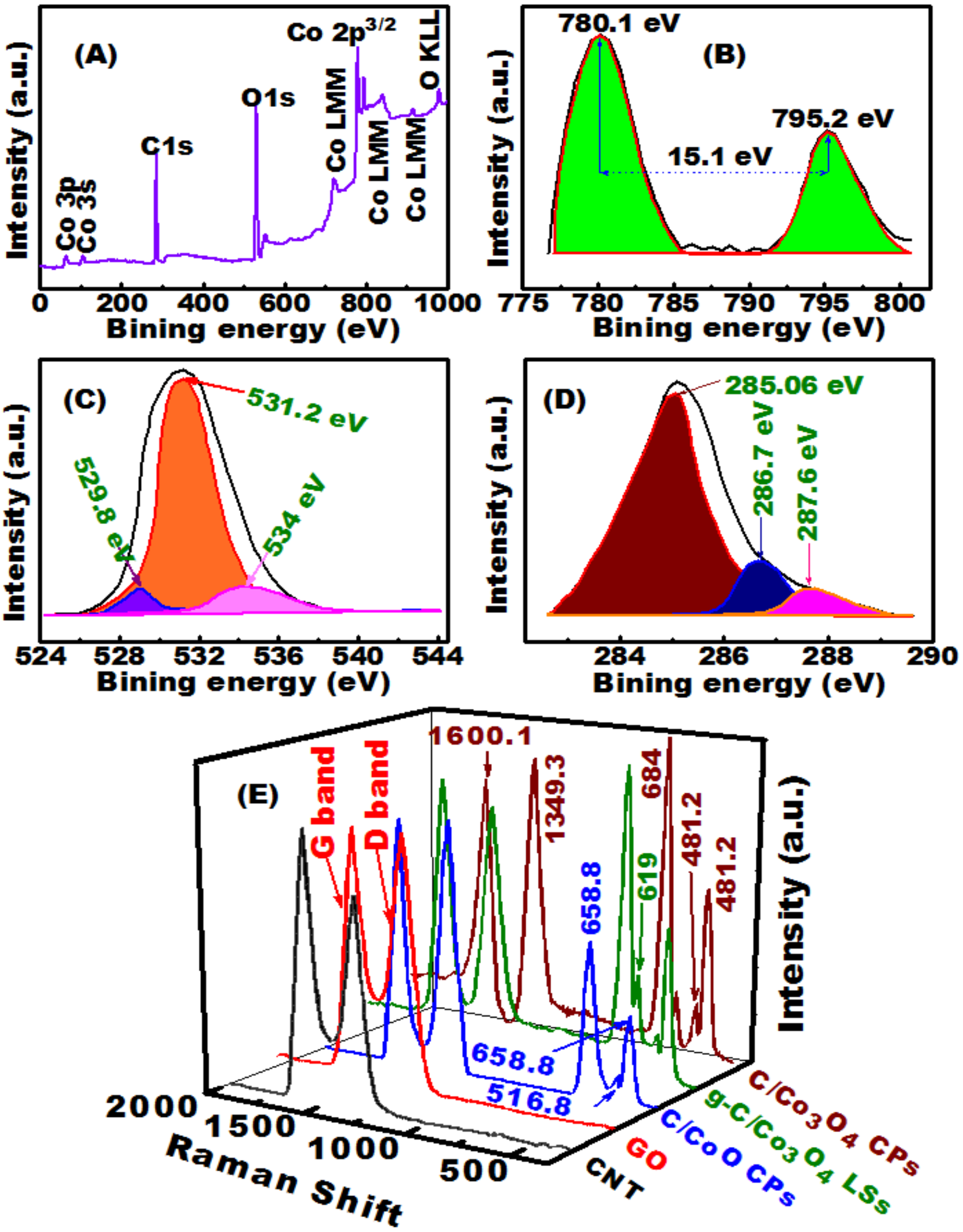

Fig. S8. (A–E) Chemical compositions and constituent states of the investigated samples measured by XPS and Raman analyses. (A) Complete survey of the XPS spectrum of C-NT/Co_3O_4 CPs showing the existence of O1s, C1s, and Co2p distinctive peaks. (B) High resolution of the Co2p peak deconvoluted into two characteristic peaks, with an energy difference of 15.0 eV. (C) High resolution C1s scan deconvoluted into three influential peaks. (D) High resolution O1s peak deconvoluted into three peaks. (E) Raman spectroscopy investigation of the samples measured at a laser beam of 633 nm.

Supplementary S9

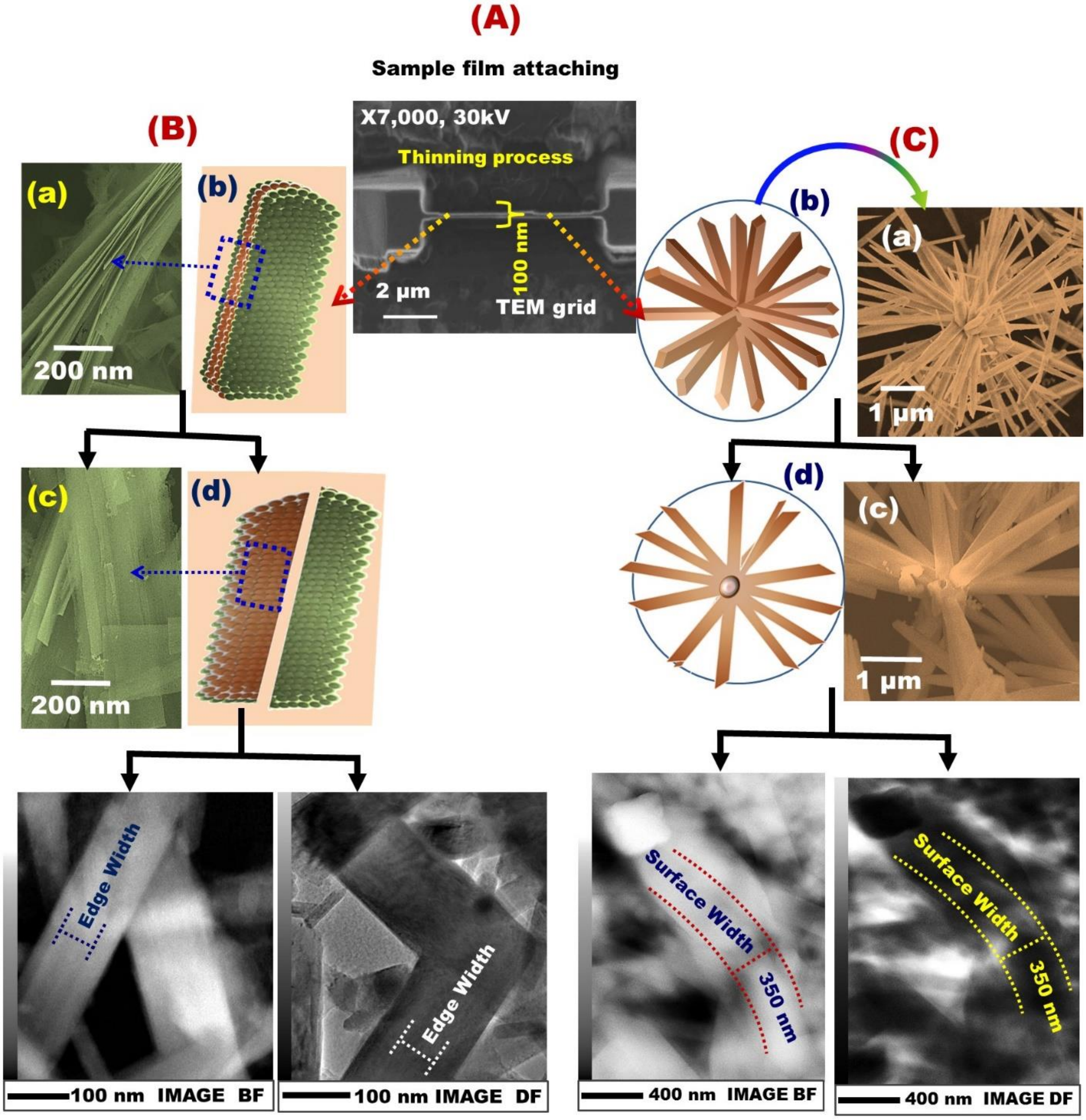

Fig. S9. (A, B-b–B-d, C-a–C-d) FIB setup showing the specimen preparation by FIB investigation before characterization by HAADF–STEM. (A) Low-magnification cross-sectional FESEM images indicating the 100 nm sample operation by the FIB system under the electron beams. (B-a–B-d, C-a–C-d) SEM and the corresponding schematic illustration of the microtomed g-C/Co_3O_4 Ls and C-NT/Co_3O_4 BCs, respectively, by etching the specimen parallel direction of longitudinal axis. The lower images are the related HAADF–STEM (bright/dark) images of the microtomed Co_3O_4/g-C Ls and C-NT/Co_3O_4 BCs specimens.

Supplementary S10

The crystallographic nature and morphological shape of the individual mesocrystals of the hierarchical C-NT/$Co(OH)_x(CO_3)_{0.5}\cdot 0.11H_2O$ BC-NRs were evident from the HAADF–STEM and ED images (inset), as shown in Figures S10A and S10B. With high-temperature treatment, no change was observed in the morphological shape of the BC-NRs (Figures S8C–S8E).

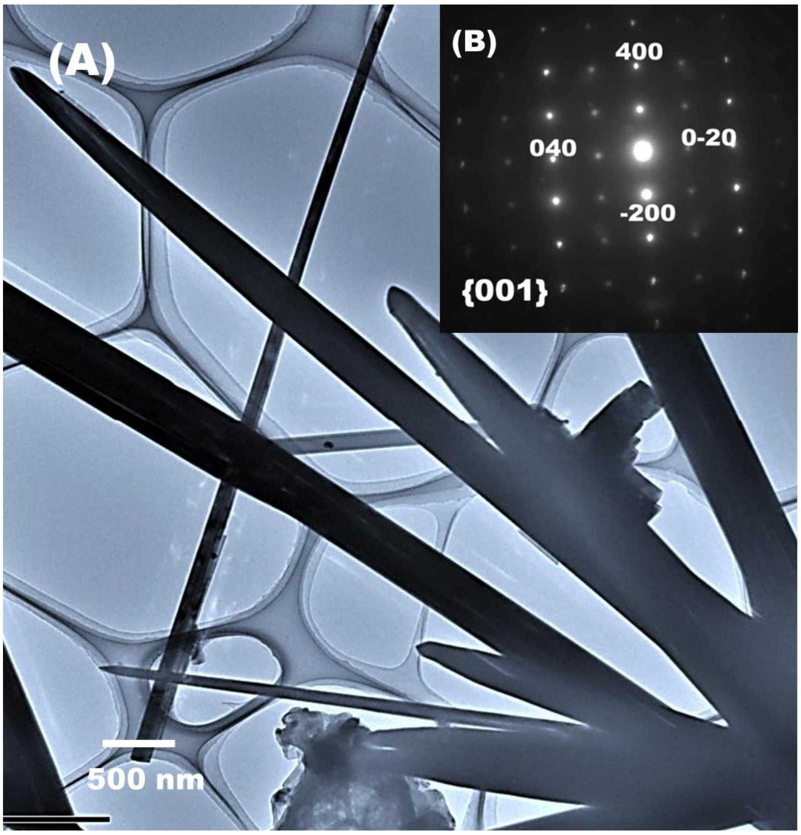

Fig. S10. (A) HAADF–STEM micrographs of hierarchical C-NT/Co_3O_4 BCs/3D PNi substrates observed along the {001} plane illustrating the corresponding electron diffraction (B).

Supplementary S11

To control the electrochemical performance of the electrodes, the cyclic voltammetry (CV) technique was initially applied in 0.5 M NaOH using a N_2-saturated electrolyte at a scan rate of 50 mV s^{-1} at room temperature (details are available in the Electrochemical Measurements segment of the Supplementary Section). Notably, the current density of well-cleaned 3D PNi was basically negligible (~0.5 mA/cm^2) compared with that of the proposed electrode designs (Figure S11A). Under pure alkaline conditions, all of the electrodes revealed two sets of redox couples produced from the reversible reactions of Co_3O_4 and CoOOH (peaks I/IV) and between CoOOH and CoO_2 (peaks III/II) (Figures 3, S11, S12, S13, S14A-D, and S15). The C-NT/Co_3O_4 CPs/3D PNi electrode exhibited the largest redox peaks among the electrodes used, indicating the high surface reactivity and accessibility of the electrode to the electrocatalytic reactions. The C-NT/CoO CPs/3D PNi electrode exhibited the lowest activity among the electrodes. This finding can be attributed to poorly developed active sites and weak electron transport in the materials used to build this electrode. To highlight the synergistic effect of the counterparts on the electroactivity of the electrodes, the activity of single Co_3O_4 or CoO/3D PNi electrodes was investigated by CV under the same conditions in 0.5 M NaOH (Figure S11A). The CVs of the Co_3O_4 or CoO/3D PNi electrodes featured redox behaviors similar to those of the C-NT or g-C/Co_3O_4 or CoO/3D PNi electrodes despite the negligible shift in the redox peak positions (Figures 3A and S13B). Improvements in electrical conductivity over the large surface coverage of electrodes from the synergetic embedding of C-NT or g-C/Co_3O_4 or CoO/3D PNi electrode designs were evident from the enhancement of the peak currents of the electrodes (Figure 3). Hence, the strong synergetic coupling between Co_3O_4 or CoO and C-NT or g-C with retention of the morphological hierarchy in the longitudinal direction may be the key determinant of high electrical conductivity and easy electron transport in the Co_3O_4 or CoO/carbon/3D PNi electrodes.

Figures S11B-C show the CV profiles of pristine g-C and C-NT counterparts in 0.5 m NaOH recorded at a scan rate of 50 mV s¯1, and with/without addition of 0.5 M ethanol. Findings show evidence that both g-C and C-NT counterparts have poor electro-catalytic activity towards EOR in alkaline conditions. In electrochemical assays with addition of ethanol, the observed CV profiles of g-C and C-NT demonstrate that no oxidation current for ethanol electrooxidation is detected as the same trend as in pure alkaline media.

To study the synergetic contribution of the g-C and C-NT counterparts to the EOR electroactivity of the hybrid electrodes, we carried out a set of electrochemical experiments (see Figure S11-S14). The CVs of pure Co_3O_4 and g-C or C/Co_3O_4 based electrodes were recorded (Figures 4A-B, S14E and S11D-E). Interestingly, the remarkable enhancement of the catalytic activity of the g-C or C-

NTs/Co_3O_4 electrodes is evident. This result indicates the significant role of the counterparts to provide a fast charge transport, to enhance the diffusion of active species along the electrode building[25,26] and to afford abundant of active sites for EOR. Moreover, the g-C or C-NTs/Co_3O_4 electrodes show higher anodic current densities and lower onset potentials than that of pristine Co_3O_4 electrodes (Figs 4A-B and S11F).

The electrochemical findings of the hierarchy g-C or C-NT/Co_3O_4/3D PNi or GC hybrids clearly show evidence of the key component of the g-C or C-NT counterparts in the enhancement of the catalytic activity of Co_3O_4 electrodes (See Supporting S11–S15). In our EOR assays, both g-C and C-NT counterparts can act as (i) a supporter for the active Co_3O_4 mesocrystal catalysts and (ii) as an electron source for providing electron mobility and movement at the electrode–electrolyte interfaces during the EOR. The high electrical properties of the g-C or C-NT counterpart improve the electronic conductivity of the hybrid catalysts and foster the electron transfer kinetics at the active sites on electrode surfaces, thereby resulting in better utilization of the Co_3O_4 mesocrystal catalysts[16,17,19]. Our finding indicates that the highly conductive g-C counterpart provides double current density and facile electron transport at the electrode–electrolyte interfaces of the C-NT support, which leads to the ultrafast electron transport kinetics of the electrooxidation reactions (See Supporting S11B&C)[16,19]. Consequently, the facile charge/electron transport through the tunneling C-tubular cylinders or ordered g-C sheet layers leads to the effective removal of intermediates and the continuous oxidation of organic molecules onto the electrode surfaces[41]. The catalytic activity of all hierarchy g-C or C-NT/Co_3O_4/3D PNi or GC hybrids is also improved indiscriminately by the synergetic role of the C supports.

The coupling of the g-C or C-NT counterparts leads to an increase in the specific surface area and a decrease in the morphological-sized structures of Co_3O_4 catalyst mesocrystals from micrometric scale to sub-nanoscale ranges (see Supporting S7), suggesting that more catalytically active coverage surfaces of the C-NT or g-C/Co_3O_4 electrodes could be exposed. Thus, the synergetic bonding and interaction between the surface-O-containing groups of C-NT or g-C and cobalt enable the well dispersion of Co_3O_4 mesocrystals in longitudinally axial building architectures that eliminate the lateral diffusion and retain the surface catalytic activity and stability of electrodes after multiple reuse/cycles.

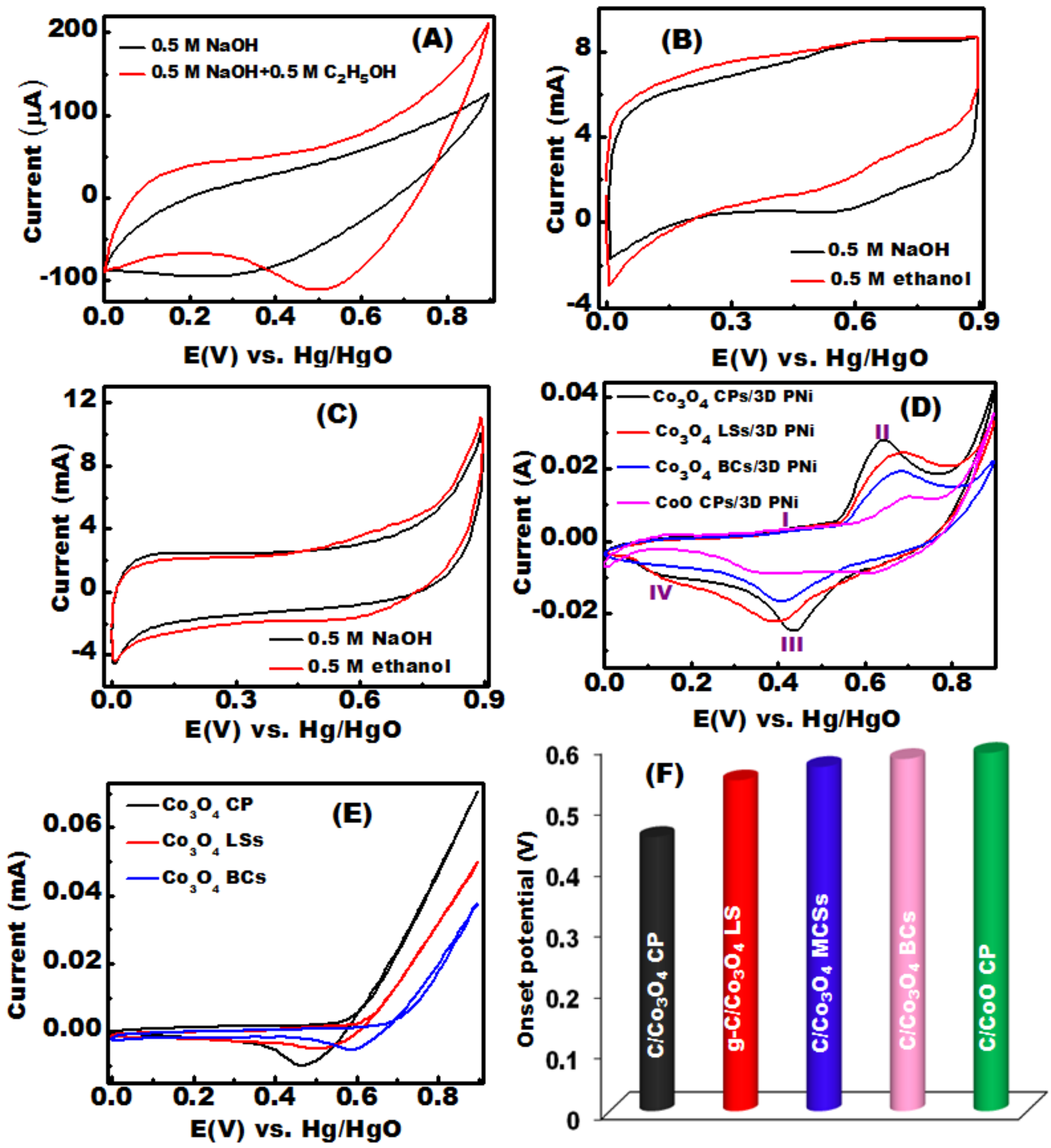

Fig. S11. (A-F) CVs of the electrodes recorded in 0.5 M NaOH at a scan rate of 50 mV s^{-1} at room temperature in absence and presence of 0.5 M ethanol. (A) CV responses of the bare 3D PNi electrodes in the absence and presence of ethanol, (B) CVs of g-C, (C) CVs of CNT, (D) CV profiles of Co_3O_4/3D PNi electrodes in 0.5 M NaOH, (E) CVs of pure Co_3O_4 electrodes in 0.5 M ethanol, and (F) the observed onset potential of the g-C or C/Co_3O_4 or CoO/3D PNi electrodes.

Supplementary S12

Figure S12 reveals the effect of scan rate on the CV response of electrodes. Particularly, we studied the CVs of C-NT/Co_3O_4 CPs/3D PNi or GC-based electrodes in 0.5 M NaOH at various scan rates from 20 mV s^{-1} to 200 mV s^{-1} (Figure S12). With increasing scan rate, the redox peak currents of both the anodic and cathodic scan increased linearly despite the –ve and +ve shifts, respectively, in both surface-controlled processes at scan rates > 50 mV s^{-1}. This result indicates facile electron transport and diffusion along the longitudinal surfaces of the electrodes[33].

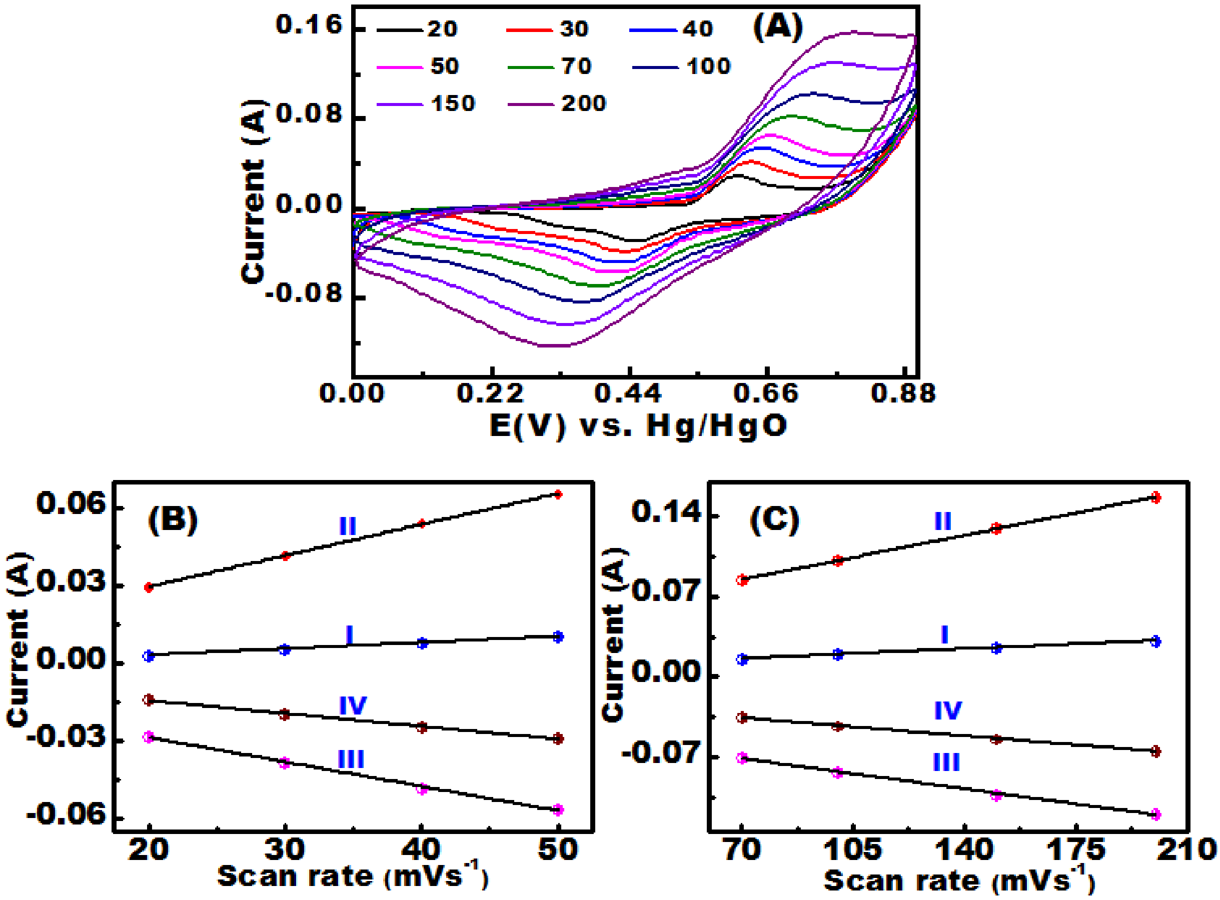

Fig. S12. (A–C) Effect of scan rate on the behavior of the C-NT/Co_3O_4 CPs/3D PNi electrode. (A) CV responses of C-NT/Co_3O_4 CPs/3D PNi electrode recorded in 0.5 M NaOH at different scan rates from 20–200 mV s^{-1} at room temperature. (B, C) Plots of peak currents vs. the applied scan rate (B) at 20–50 mV s^{-1} and (C) at 70–200 mV s^{-1}.

Supplementary S13

To further illustrate the superior effect of the direct longitudinal growth of electroactive C/Co_3O_4 catalysts into free-supporting 3D PNi electrode on electrochemical activity in terms of current density and stability of electrodes, we performed the EOR using a C-NT or g-C/Co_3O_4/GC electrode (Figures S13–S15). The GC-based electrode was fabricated through a heterogeneous-assisted ink deposition method (see Experimental Section in Supplementary Information). A set of EOR catalytic experiments was also performed using both electrodes based on C-NT or g-C/Co_3O_4 and C-NT/CoO CPs to show the effective influence of Co^{3+} site composition on EORs.

Similar to the electroactivity of the 3D PNi modified electrodes in the absence or presence of ethanol, the catalytic activity of the GC-based electrodes was improved indiscriminately by the synergetic role of the counterparts (Figure S15). However, the disparity in catalytic activity (Figure S13) among the electrodes can be ascribed to the interactions of the catalytically exposed sites. Particularly, the interplay of Co^{3+} with the counterparts added significant advantages to the catalytic activity for the EOR. A higher change in the relative current of GC-based electrodes was observed after long-term cycling (i.e., 18,000 s) relative to that of the free-supporting 3D PNi -based electrodes. This finding indicates the efficient design and stability of the PNi-based electrode (Figures S13-S13). The results also reveal the beneficial and fractal effects of the direct, longitudinal growth of carbon/Co_3O_4 into free-supporting 3D PNi electrodes from the vertical alignment of NR, MCS, and LS morphologies. Their robustness in the electrode design into open and connective macroporous pores of PNi carriers ensured much higher molecular trapping, diffusion levels, and facile electron movements along this unique longitudinal build.

Our findings show a systematic electron movement concept along the LSs and MSCs could be manifested from the catalytic performances of both electrodes (Figures 3 and S13). The LS current density was mostly higher than that of the MCSs, indicating that the catalytic efficiency of LSs and MCSs can vary in several key parameters as follows: (i) the nanoscale thickness of the edge and length of fattened lamina-layers, (ii) the vertically one-directional, alternately parallel, and discrete layers leading to symmetrically homogenous electron diffusivity among laminas, and (iii) the minimal electron movement across a longitudinal ridge channel in the rough MCSs (coherently opposite double-helix designs). In the design of MCSs, the effect of the position of the lamina multilayers lying opposite one another, with connecting horns along the central or longitudinal ridge, could reinforce the instability of electron transport through the ridge gate. Each opposing lateral across the contact ridge could also hinder or suppress the electron diffusivity to all the layers and areas once situated in both sides of the MCSs.

Among the electrodes, the C-NT/Co_3O_4 CP electrode exhibited the highest activity, suggesting more exposed active sites in this electrode, which indicates that numerous Co_3O_4 mesocrystals have participated effectively in the reaction.

The lower onset potential of the carbon/Co_3O_4 electrode with respect to those of other electrodes denotes the lower overpotential and superior kinetics for the EOR. Moreover, the relatively high oxidation current and lower onset potential demonstrate the higher catalytic activity of the electrode towards EOR. On the other hand, the C-NT/CoO electrode mainly contains a wide range of Co^{2+} active sites that are inactive for EOR. The lower electroactivity and slow kinetics of the C-NT/CoO CP electrode can be explained by its lower oxidation current and higher positive onset potential.

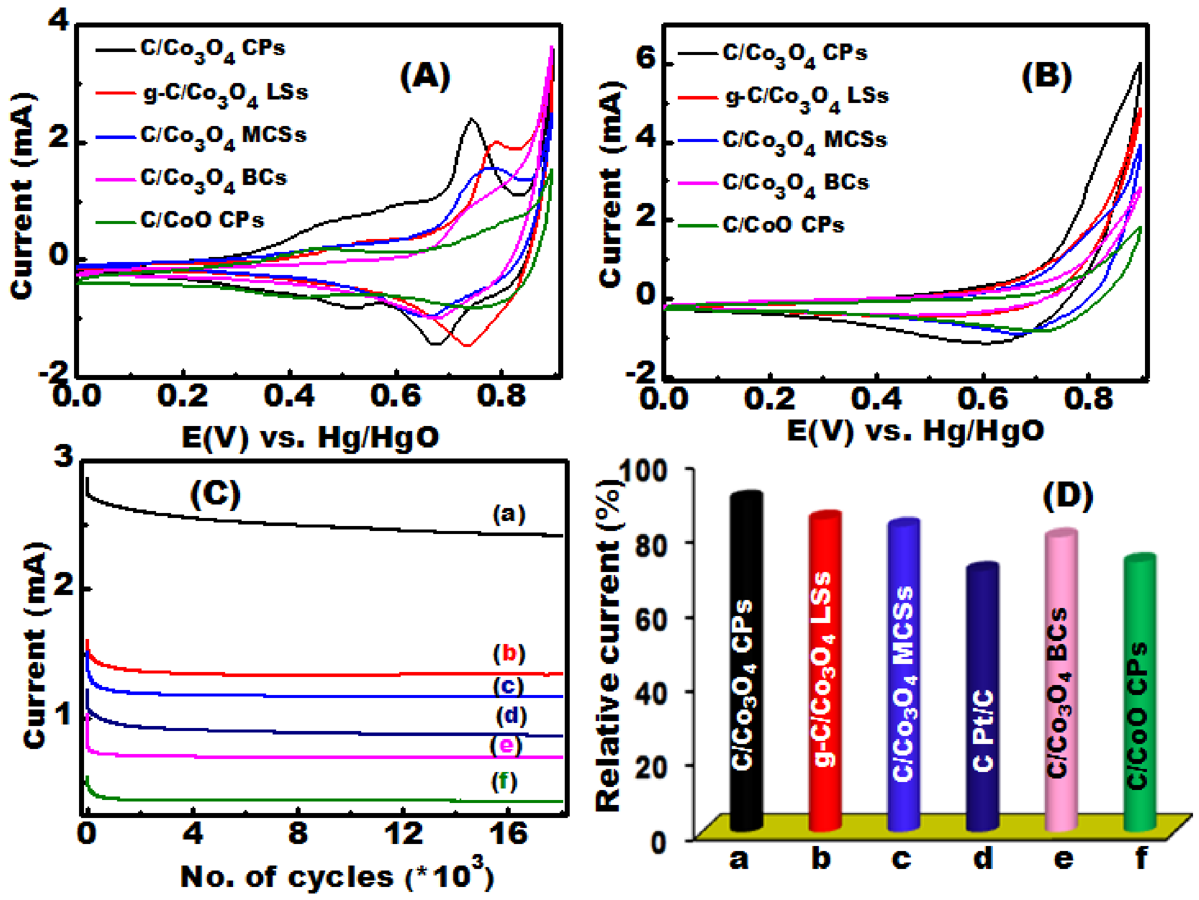

Fig. S13. (A–D) Electrochemical evaluation of carbon/Co_3O_4 or CoO/GC electrodes in 0.5 M NaOH and N_2-saturated electrolyte in the absence or presence of ethanol at a scan rate of 50 mV s^{-1}. CV profiles recorded in the absence (A) and presence (B) of 0.5 M ethanol. (C) Current–time relationships of the carbon/Co_3O_4 or CoO /GC electrodes in 0.5 M ethanol for 18,000 s. (D) Relative currents of g-C or C-NT/Co_3O_4 or CoO/GC electrodes as a function of the initial current at the start of the CA test. Note that a, b, c, d, e, and f represent C-NT/Co_3O_4 CPs/GC, g-C/Co_3O_4 LSs/GC, C-NT/Co_3O_4 MCSs/GC, commercial Pt/C electrode, C-NT/Co_3O_4 BCs/GC, and C-NT/CoO CPs/GC electrodes, respectively.

Supplementary S14

EOR measurement of C-NT/Co_3O_4 **CPs/GC electrode**

The electrochemical performances of the C-NT/Co_3O_4 CPs/GC modified electrode were evaluated by CV (Figures S14–S15) in 0.5 M NaOH in the absence and presence of ethanol. By considering the same active mass of the C-NT/Co_3O_4 CP catalyst uniformly loaded on both substrates, the C-NT/Co_3O_4 CPs/3D PNi conductive substrate creates a cell capable of multiplying the efficiency of EOR to more than three times higher in current density than the film-casted/GC-based electrode (Figure S14). The marked currents of the C-NT/Co_3O_4 CPs/3D PNi electrode is mainly due to the direct contact of the active material to the current collector (3D PNi), which ensures fast electron transport. Moreover, the absence of polymeric binders can greatly improve the electrode's electrocatalytic activity.

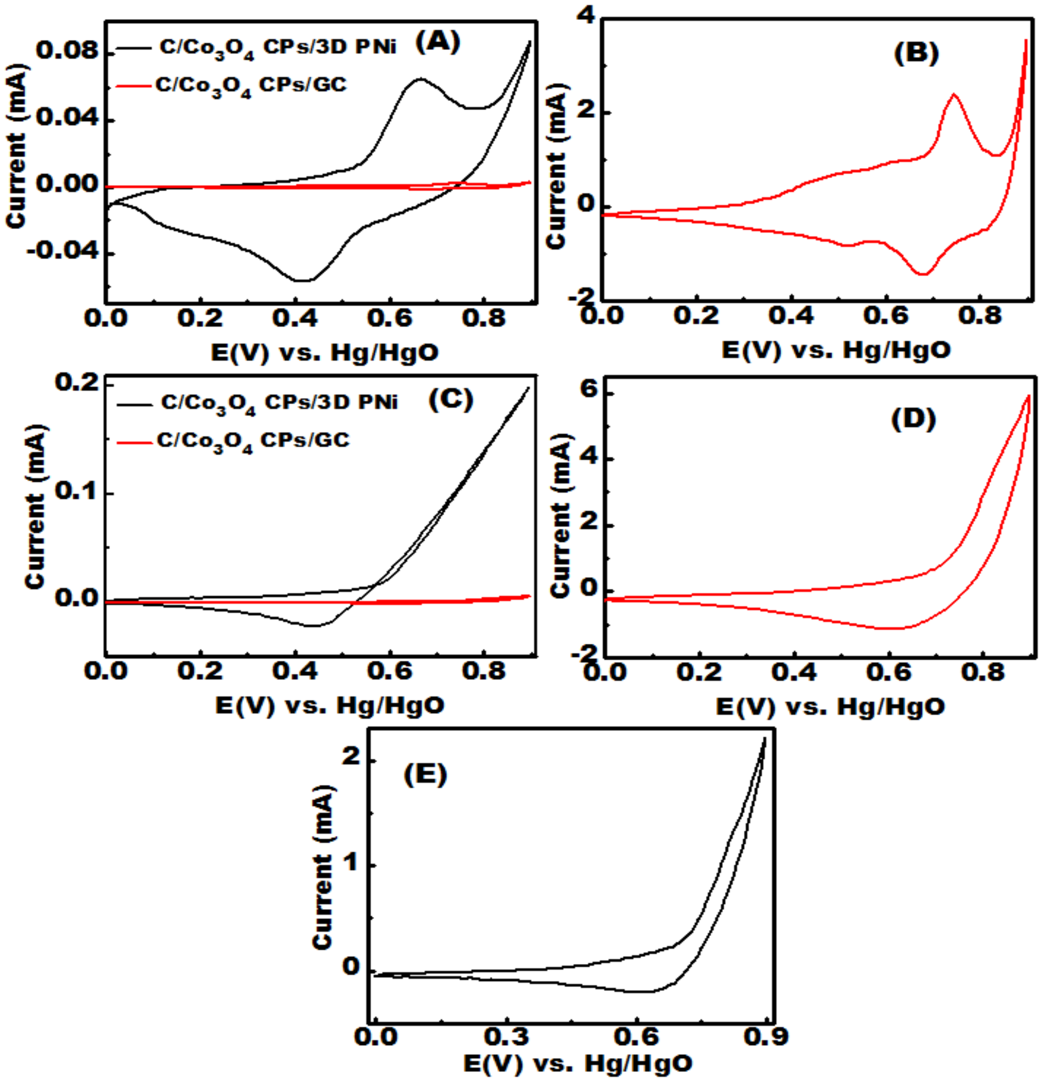

Fig. S14. (A–D) CV curves of C-NT/Co_3O_4 CPs/GC and C-NT/Co_3O_4 CPs/3D PNi electrodes recorded in 0.5 M NaOH at 50 mVs^{-1} and at room temperature. (A) CVs of C-NT/Co_3O_4 CPs/GC and 3D PNi electrodes in the absence of ethanol at 50 mV s^{-1} scan rate. (B) Magnified CV spectra of C-NT/Co_3O_4 CPs/GC in the absence of ethanol at 50 mV s^{-1} scan rate. (C) CVs of C-NT/Co_3O_4 CPs/GC and 3D PNi electrodes in the presence of 0.5 M C_2H_5OH at 50 mV s^{-1} scan rate. (D) High-resolution CV spectra of C-NT/Co_3O_4 CPs/GC electrode in the presence of 0.5 M C_2H_5OH. (E) CV response of bare Co_3O_4 CP/GC recorded in 0.5 M ethanol.

Supplementary S15

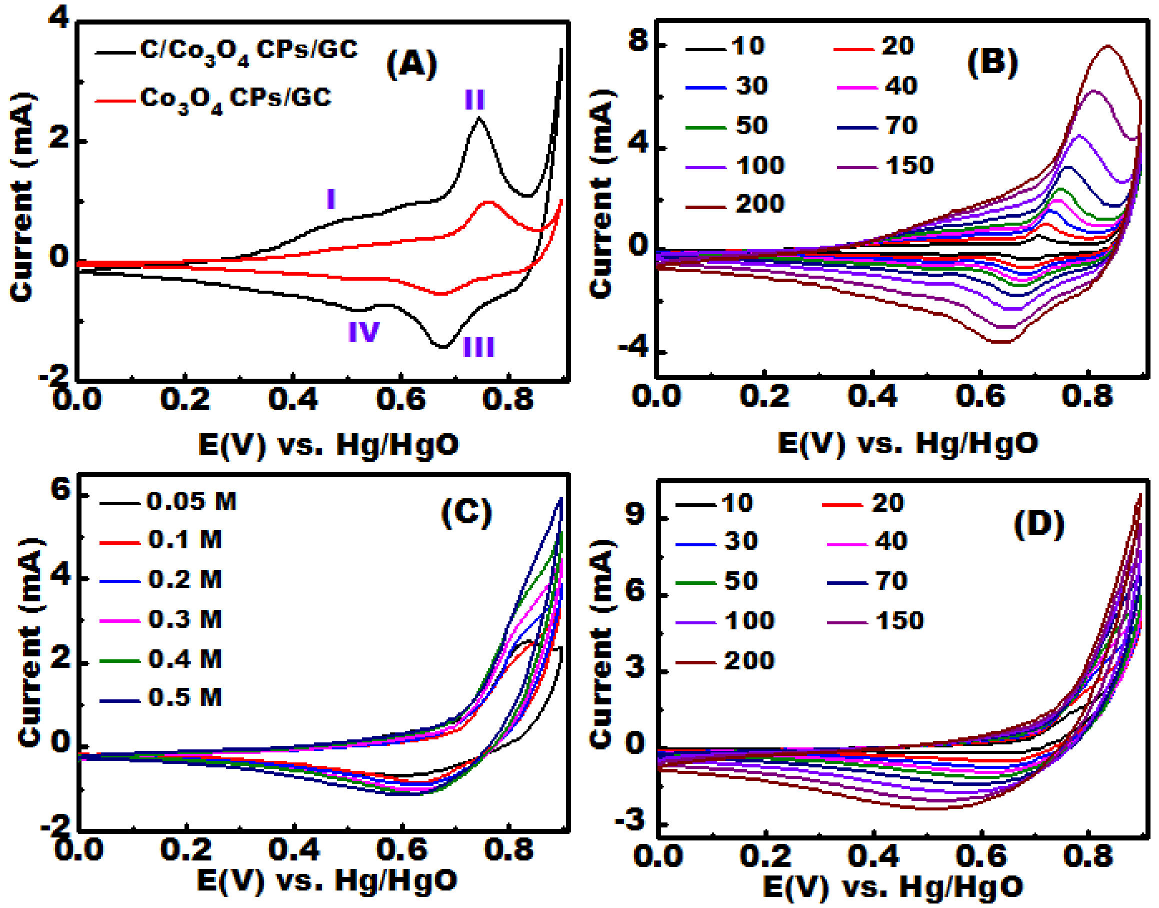

Fig. S15. (A–E) CVs of C-NT/Co_3O_4 CPs/GC modified electrodes recorded in 0.5 M NaOH at room temperature. (A) CVs of C-NT/Co_3O_4 CPs and bare Co_3O_4 CPs/GC-based electrodes collected in 0.5 M NaOH solution after100 sweeps at a scan rate of 50 mV s^{-1}. (B) CV plots of C-NT/Co_3O_4 CPs/GC electrode recorded in 0.5 M NaOH at various scan rates. (C) Effect of ethanol concentration on the CV responses of the C-NT/Co_3O_4 CPs/GC electrode at 50 mV s^{-1} scan rate. (D) CV behaviors at different scan rates measured in 0.5 M NaOH.

Supplementary S16

Effects of ethanol concentration and scan rate on the performance of C-NT/Co_3O_4 **CPs/3D PNi electrode**

In this electrochemical assay, several key factors, including ethanol concentration and scan rate, significantly affected the EOR activity (Figures S16 and S15). Concentrated ethanol solution is known to be suitable for use in DEFCs to minimize their size. In this study, ethanol concentration was changed from 0.05 M to 0.5 M. Figure S16A and S15 presents the CV profiles of C-NT/Co_3O_4 CPs/3D PNi electrode measured at various ethanol concentrations.

The ethanol oxidation current increases as the ethanol concentration increases, indicating that the oxidation of ethanol molecules at higher potentials leads to the formation of highly active Co(III) species[30] (equations 1, 2). The linear change of the oxidation current with increasing ethanol concentration shows the homogenous formation of CoOOH-to-ethanol layers with facile diffusion and electron transport (Figure S16B). With high doses of ethanol (i.e., > 0.5 M, data not shown), the electrode reaction surfaces reaches saturation, indicating the difficulty to achieve faster electron transport along the electroactive layers in this case.

$$Co(III) \leftrightarrow Co(IV) + e^{-} \quad (1)$$

$$Co(IV) + ethanol \leftrightarrow Co(III) + product \quad (2)$$

To study the behavior of the electrode, the CV was investigated over a scan sweep of 10–200 mV s^{-1} in 0.5 M ethanol (Figures S16C-E and S15D). The increase in scan rate also increased both cathodic and anodic currents, particularly at scan rates > 50 mV s^{-1}. The linear dependency of the ethanol oxidation current on the square root of the scan rate (Figure S16D) indicates that the oxidation of ethanol is a diffusion-controlled process (Scheme 2). An increase in the cathodic peak current of the electrodes (i.e., C-NT/Co_3O_4 CP/3D PNi electrode) was noted with increasing ethanol concentration and scan rate (i.e., > 50 mV s^{-1}). This finding may be due to the inability of the electrode to reduce the high valence of the Co(IV) species formed on the surfaces enriched with high-index single crystal {112} and interface {111}/{112} planes during anodic sweep at high ethanol concentrations and scan rates[31, 32]

The gradual increase in cathodic peak with a slightly negative shift with increasing scan rate suggests that the EOR in the cathodic path is scan-rate dependent. This trend shows that ethanol oxidation of the higher-valence metal oxides is a rate-determining process, as reported by Fleishmann et al.[33]. The ratio of the anodic to cathodic current (Ia/Icat) (Figure S16E) is high at low scan rates, which might

be due to the rapid formation of the active CoOOH layer. The change in the Ia/Ic ratio is approximately negligible after a sweep rate of 100 mV s^{-1}.

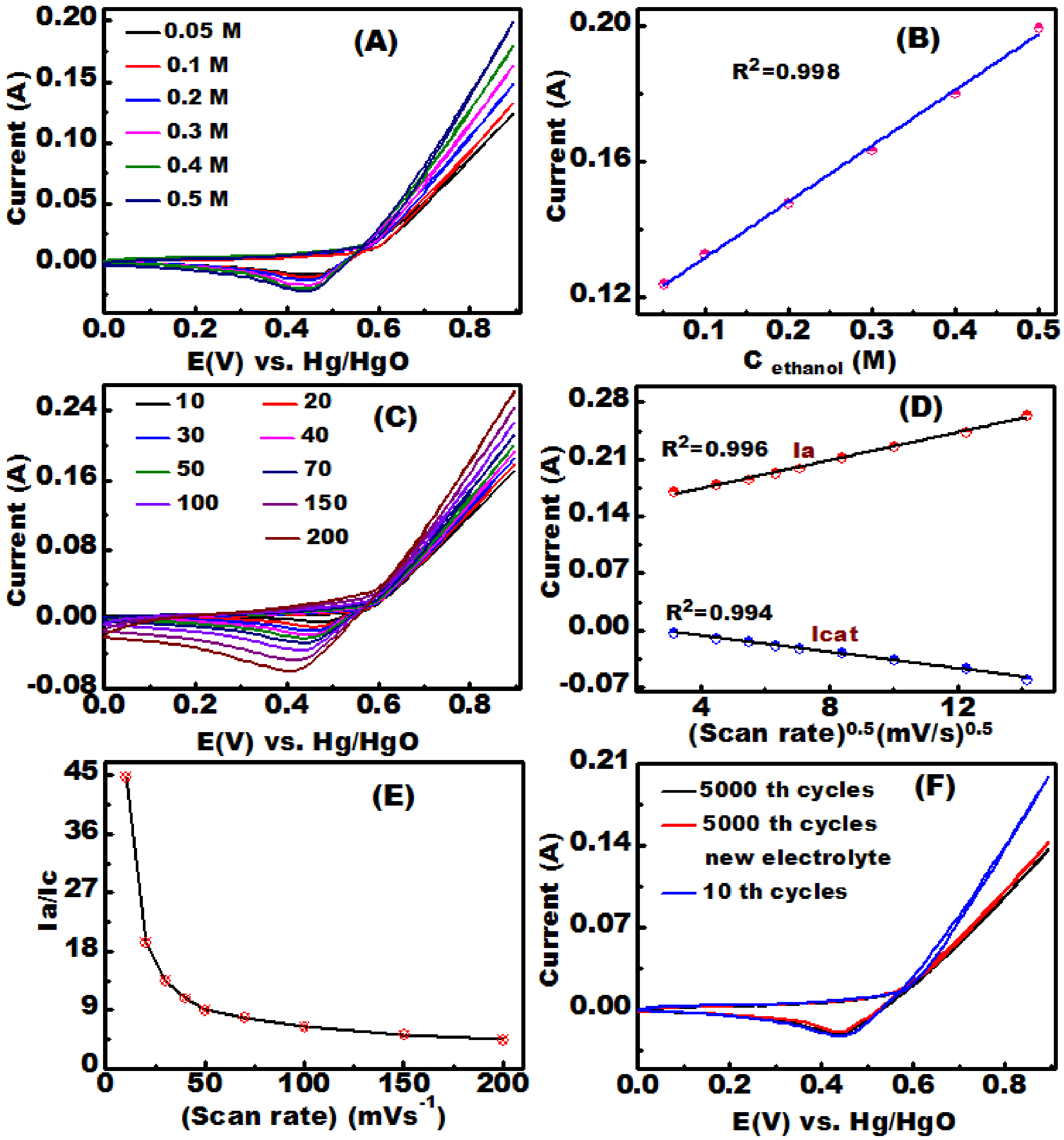

Fig. S16. CVs of C-NT/Co_3O_4 CPs/3D PNi electrode recorded in 0.5 M NaOH at room temperature. (A) CVs recorded at various concentrations of ethanol (0.05–0.5 M) and a scan rate of 50 mVs^{-1}. (B) Relationship between ethanol concentration and the corresponding current density measured at the end of the anodic scan. (C) CV responses of the C-NT/Co_3O_4 CPs/3D PNi electrode at different scan rates from 10–200 mV s^{-1} obtained in 0.5 M NaOH containing 0.5 M ethanol. (D) Plot of the catalytic currents (Ia, Ic measured at the end of forward scan and at the cathodic peaks) *vs.* the square root of the applied scan rate. (E) Plot of Ia/Ic *vs.* the applied scan rate. (F) CV curves of C-NT/Co_3O_4

CPs/3D PNi electrode collected in 0.5 M NaOH and 0.5 M ethanol after continuous potential cycling at a sweep rate of 50 mV s^{-1}.

Supplementary S17

EOR stability evaluation by CA

To highlight the advantage of the self-supported carbon/Co_3O_4 or CoO/3D PNi electrodes, the stability of the electrodes in EOR was investigated. This goal was achieved by obtaining current–time measurements using the CA technique at an applied potential of 0.7 V versus Hg/HgO in an electrolyte containing 0.5 M NaOH and 0.5 M ethanol. During the initial stage, a current drop was observed for all electrodes, followed by gradual decay, until pseudo-steady state was approached. This trend may be attributed to the accumulation of ethanol intermediates and chemisorbed species at the electrode surface, such as CO_{ads} and CHO_{ads}, that poison the electrodes. Despite the current decay, the C-NT/Co_3O_4 CPs/3D PNi electrode exhibited the highest current among all the electrodes tested, demonstrating its superior stability.

The catalytic rate constant was measured in accordance with the reported equations (3, 4) as follows[34]:

$$I_C/I_L = \gamma^{0.5}[(\pi^{0.5})erf(\gamma^{0.5}) + exp(-\gamma)(\gamma^{0.5})] \qquad (3)$$

where I_C is the catalytic current of C-NT/Co_3O_4 CPs/3D PNi in the presence of ethanol measured from the current–time spectra (Figure 3C-a), I_L is the limiting current in the absence of ethanol measured from the current–time data (Figure S17A), γ is the error function and equal to kC_ot, and C_o is the ethanol concentration. As the value of γ exceeds 2, the error function will be equal to 1, and subsequently the above equation can be reduced to:

$$I_C/I_L = \gamma^{0.5}(\pi^{0.5}) = (\pi^{0.5})\,(kC_o t^{0.5}) \qquad (4)$$

where k, C_0, and t are the catalytic rate constant ($cm^3\ mol^{-1}\ s^{-1}$), ethanol concentration ($mol\ cm^{-3}$), and time duration (s), respectively. The value of k was evaluated from the slope of the I_C/I_L versus $time^{1/2}$ graph. It is a measure of the kinetics of EOR at the electrode.

Figure S17B shows the I_C/I_L plot at C-NT/Co_3O_4 CPs/3D PNi electrode. Results show that the average value of k was found to be 4.56×10^3 $cm^3\ mol^{-1}\ s^{-1}$, indicating the high EOR kinetics. Furthermore, the linear dependence of the net currents I_C (Figure S17C) and I_L (Figure S17D) on the inverse of the root square of elapsed time ($s^{-1/2}$) reveals that the process is dominated by diffusion.

To shed light on the diffusion of ethanol molecules from the electrolyte to the catalytically active sites of the electrode, the diffusivity of ethanol molecules into the longitudinal open-pore architecture (CPs and BCs), vertically inter-layered spaces and interfaces (LSs and MCSs), and along the surface coverage and exposure sites could be calculated using the Cottrell equation (5)[35]:

$$I_t = nFAD^{0.5}C\pi^{-0.5}t^{-0.5} \qquad (5)$$

where I_t, F, and A are the transient current in the presence of ethanol, Faraday constant (96485.3365 C/mol), and geometrical surface area of the electrode (~1 cm^2 in this study), respectively. By contrast D, C, and t refer to the diffusion coefficient (cm^2 s^{-1}), effective ethanol concentration (M), and elapsed time duration (s), respectively. Our findings illustrate that the relationship of I_C versus $t^{-1/2}$ follow Cottrellian behavior. Furthermore, the linear dependency of the transient current (I_C) on the inverse of root square of time ($s^{-1/2}$) (Figure S18A–D) proves that the EOR at the electrodes is a diffusion-controlled process.

The diffusion coefficients of ethanol into the carbon/Co_3O_4 or CoO/3D PNi electrodes were calculated (Table S2) based on the Cottrell formula (3) and current–time relationships (Figure 3C-a–C-e).

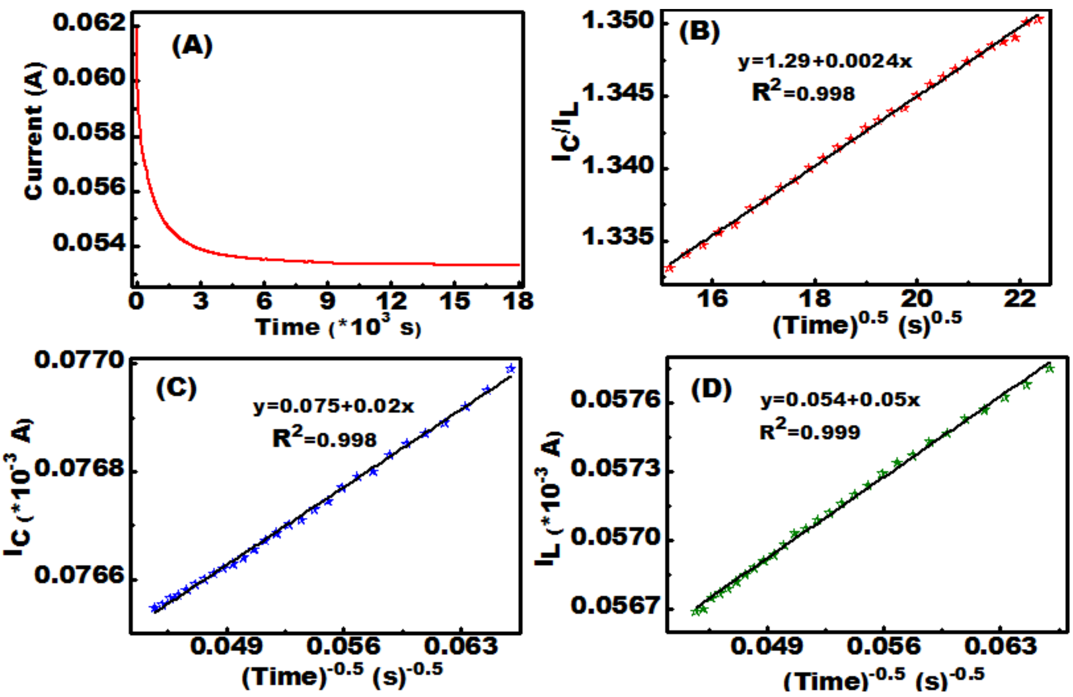

Fig. S17. (A–D) CA analyses of C-NT/Co_3O_4 CPs/3D PNi electrode evaluated in 0.5 M NaOH solution, (A) current-time spectra of C-NT/Co_3O_4 CPs/3D PNi electrode collected in absence of ethanol with a constant applied potential of 0.67 V *vs*. Hg/HgO for 1800 s (B) the relation between the (I_C/I_L) and the square roots of time applied measured from the CA date (Figure 3C-a), (C) the dependency of the catalytic current (I_C current in presence of ethanol) on the inverse of the square roots of time investigated measured from the CA date (Figure 3C-a), (D) the dependency of the limiting current (I_L current in absence of ethanol) on the inverse of the square roots of time investigated measured from the CA data (Figure S17A).

Supplementary S18

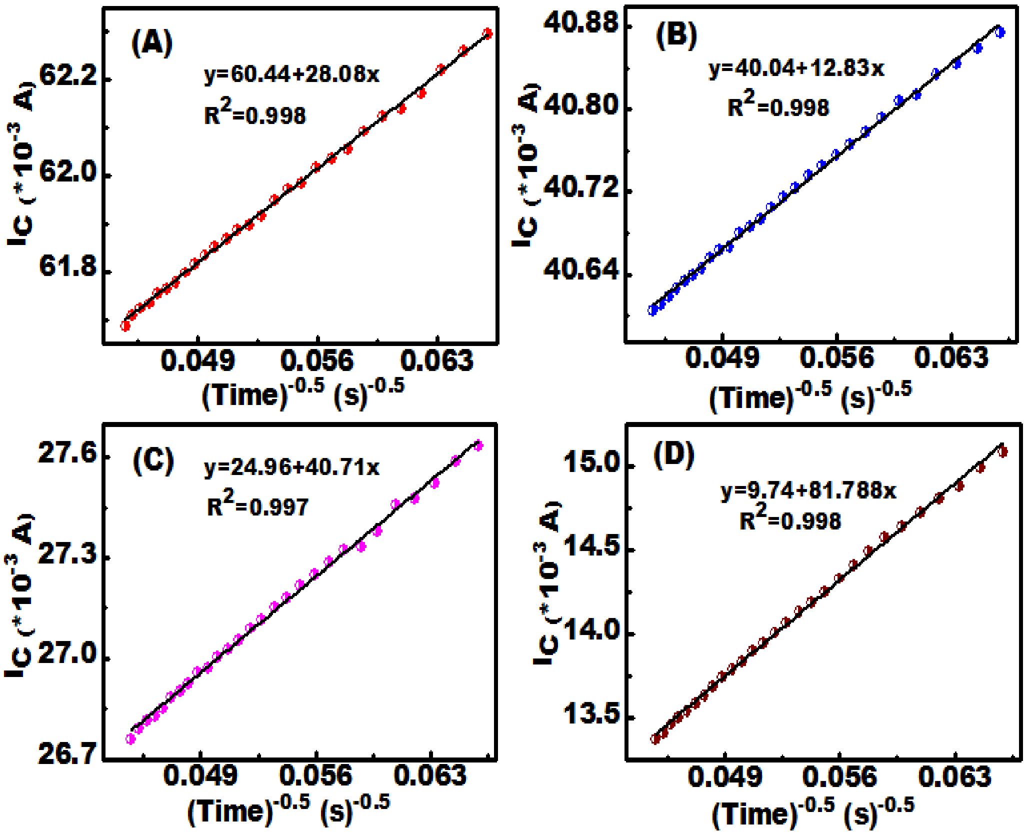

Fig. S18. (A–D) Dependence of the catalytic current (I_C) of the applied electrodes on the inverse of the square roots of time measured from the CA data (Figure 3C, curves b–e) (A) g-C/Co_3O_4 LSs, (B) C-NT/Co_3O_4 MCSs, (C) C-NT/Co_3O_4 BCs, and (D) C-NT/Co_3O_4 CPs/3D PNi electrodes.

Table S2 Measured energies of the exposed facets and interface surfaces obtained by DFT modeling.

Morphology	Single plane and interfaces	Co^{3+} density on top	Adsorption energy (E_{ads}) kJ/mol	Stabilization energy (E_B) (eV)	Surface area $(Å)^2$	Surface energy (J/m^2)	Mean diffusion Coefficient (D) ($cm^2\ s^{-1}$)
C-NT/Co_3O_4 CPs	{112}	3	-24.886	-107.155	201.19	-8.533	4.59×10^{-5}
	{111}	1	-25.845	-104.291	65.80	-23.9387	
	{111}/{112}	4	-28.472	-114.735	298.62	-6.156	
g-C /Co_3O_4 LSs	{112}	3	-24.886	-107.155	201.19	-8.533	3×10^{-5}
	{111}	1	-25.845	-104.291	65.80	-23.9387	
	{111}/{112}	4	-28.472	-114.735	298.62	-6.156	
C-NT/Co_3O_4 BCs	{001}	3	-26.028	-107.875	85.71	-20.1652	0.5×10^{-5}
	{110}	3	-26.385	-108.234	117.22	-14.794	
	{001}/{110}	2	-28.331	-101.961	212.69	-7.681	
C-NT/Co_3O_4 MCSs	{112}	3	-24.886	-107.155	201.19	-8.533	1.29×10^{-5}
	{111}	1	-25.845	-104.291	65.80	-23.9387	
	{111}/{112}	4	-28.472	-114.735	298.62	-6.156	

Supplementary S19

Stability of the hierarchical structures of electrodes with reuse/cycles

To explore the long-term stability of the longitudinal electrode designs after multiple reuse/cycles, the hierarchical structures, orientational surface crystals, and morphological shapes of the g-C or C-NT/Co_3O_4/3D PNi electrodes were investigated by HAADF-STEM (Figure S19). Figure S19 shows evidence that the hierarchical BC structures, for example, were well preserved without any changes after multiple reuse/cycling. This finding was demonstrated by the retention of the morphological BC hierarchy and the surface crystal facet orientation along the {001} planes, respectively. The C-NT/Co_3O_4 BCs (A–C) clearly exhibit a dense construction of NPs with rounded edges and clear atomic structure of {001} with crystal fringes of 2.87 Å. The surprising stability of the electrodes could be attributed to the strong interactions of the active catalyst materials with the 3D PNi substrate.

Table S3 shows evidence that the superior retention of the current density and steady-state binding response over long-term cycling observed in the C-NT/Co_3O_4 CPs/3D PNi electrode assays demonstrate the effectiveness of the electrode's properties. Furthermore, the EIS measurements show that among all the designed electrodes in this study, the C-NT/Co_3O_4 CPs/3D PNi electrode showed smaller semicircle diameters than the other electrodes, as shown in Figure 3F. The highly exposed energy surface interfaces longitudinally aligned along NRs, LSs, and MCSs considerably reduce internal resistance, provide continued pathways for electron/ion transfer, and subsequently increase the redox reaction rate. These advantages contribute to the fast kinetics of electron transport, high catalytic activity, and durability with multiple cycles that are required for DEFCs (Figure 3 and Supplementary Sections S13, S14 and S16–S18).

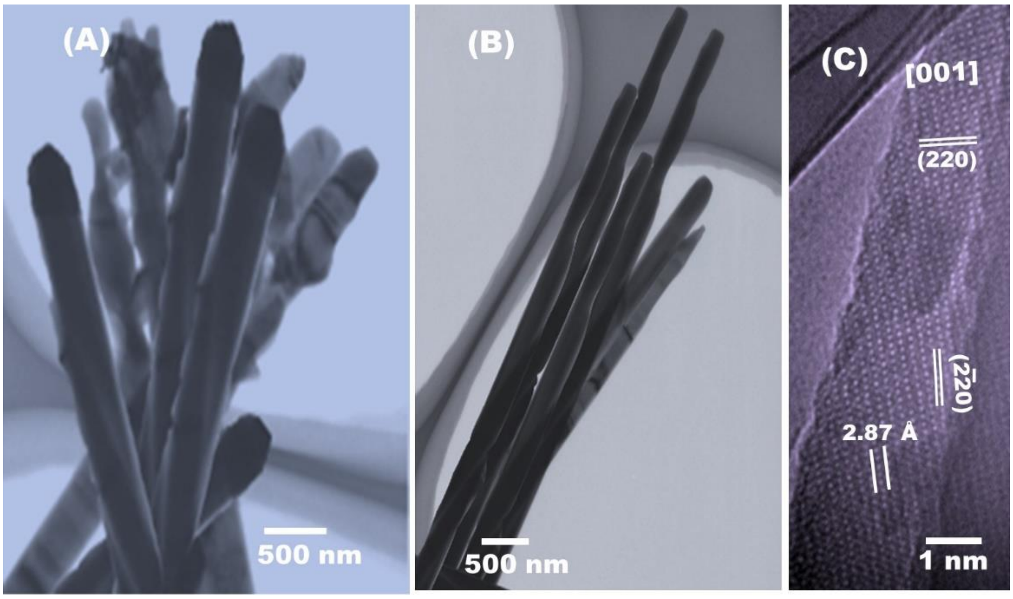

Fig. S19. (A–C) HAADF-STEM images of C-NT/Co_3O_4 BCs/3D PNi electrodes after multiple reuse cycles. The images show that the engineered morphologies of both self-supported electrodes are well preserved. (A–C) HAADF-STEM micrographs of C-NT/Co_3O_4 BCs/3D PNi measured along the {001} plane display the engineered structure after stability testing for 18000 s. (A, B) STEM micrographs illustrate the surface morphology of the NRs at different locations. (C) HR-HAADF-STEM image shows the atomic structure down {001}.

Table S3 Retained oxidation currents as percentages of their original values after long-term stability assessment of the different electrode materials relative to the C-NT/Co_3O_4 CPs/3D PNi electrode.

Active material	Retention percentage of the original value after long term stability	Effective No. of cycles	Target fuel	reference
$NiCo_2O_4$	89%	500	methanol	36
$NiCo_2O_4$	90% and 88% for NCO-NS and NCO-NC	1000	methanol	37
Go- $NiCo_2O_4$	79.3%	500	methanol	38
$NiCo_2O_4$	72% with SDS and (75%) without SDS	1000	methanol	39
$NiCo_2O_4$	85%	500	methanol	40
C/Co_3O_4/3D PNi substrate	71.9%	5000	ethanol	this study

Supplementary S20

Proposed mechanism of EOR

Basically, the proposed mechanism of EOR can be interpreted and analyzed on the basis of the adsorption phenomena of ethanol molecules and the reaction kinetics on the anisotropic surfaces (Scheme 2). In this EOR process, the oxidation of ethanol occurs in multiple steps and may generate 12 electrons. Specifically, the oxidation of ethanol at the electrode creates many oxidative derivatives that contain numerous types of chemical bonds, such as the C–C, C–O, O–H, and C–H bonds[41,42]. Numerous kinds of products are formed from the oxidation of ethanol, including acetaldehyde, acetic acid, Co, and CO_2, in addition to other adsorbed molecules like methane and ethane, as well as CO adsorbate. H_2O or OH adsorbate is a key step for the complete oxidation of ethanol to CO_2. These multiple products require the utilization of extra oxygen atoms during ethanol oxidation[41,42]. Scheme 2 shows evidence that the pathways of EOR is possibly dependent on the accessibility of the active sites. Thus, ethanol can react directly with adsorbed water or hydroxyl species and produce CO_2 over the free active sites (Figure S20A-D) into the longitudinal, interlayered, and surface directions. The reaction intermediates (e.g., CO) may have formed through the dissociation of chemisorbed molecules onto the electrode surface, consisting mainly of CO molecules. This occurrence may lead to the blocking of active sites and surfaces and the consequent reduction in electrode performance. The decay of the oxidation current (Figures 3C and S13C) with time may be attributed to the electrode surface poisoning by the strongly adsorbed species, which also indicates the reaction kinetics. In our electrochemical assays, the reduction of the poisoning species formed on the electrodes can be controlled at low potential experiments <<0.9 V. Thus, the enhanced kinetics of EOR using the designed electrode is directly demonstrated by the catalytic functionality of the electrodes. Significantly, the continuous production of OH during ethanol oxidation is the key step for the removal of CO_{ads}.

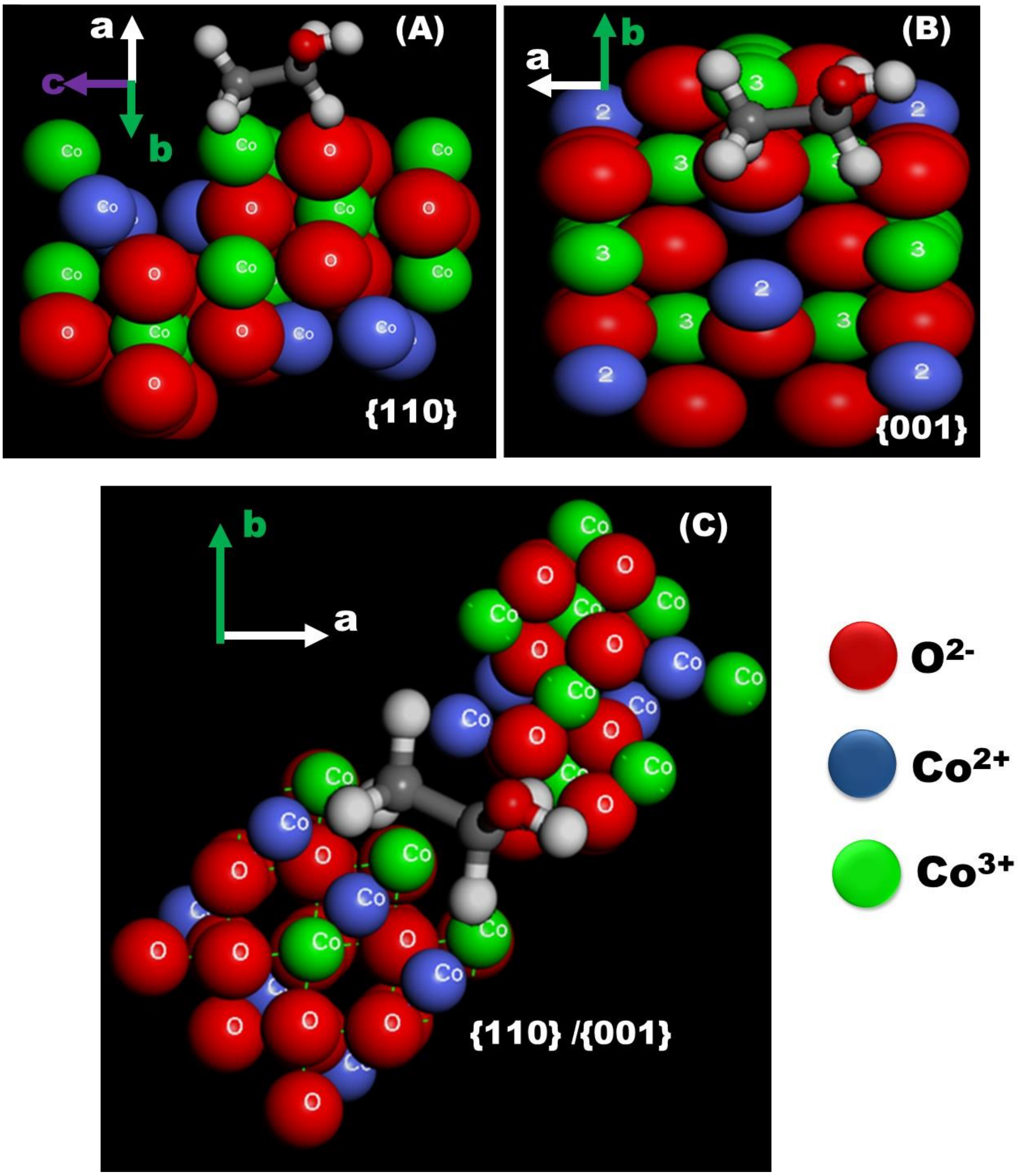

Fig. S20. (A–C) Schematic representation of Co_3O_4 crystal facets. (A-C) Simulation model of the C/Co_3O_4 CBs along the (A) {110}, (B) {001} and (C) {110}/{001} projections.

Supplementary S21

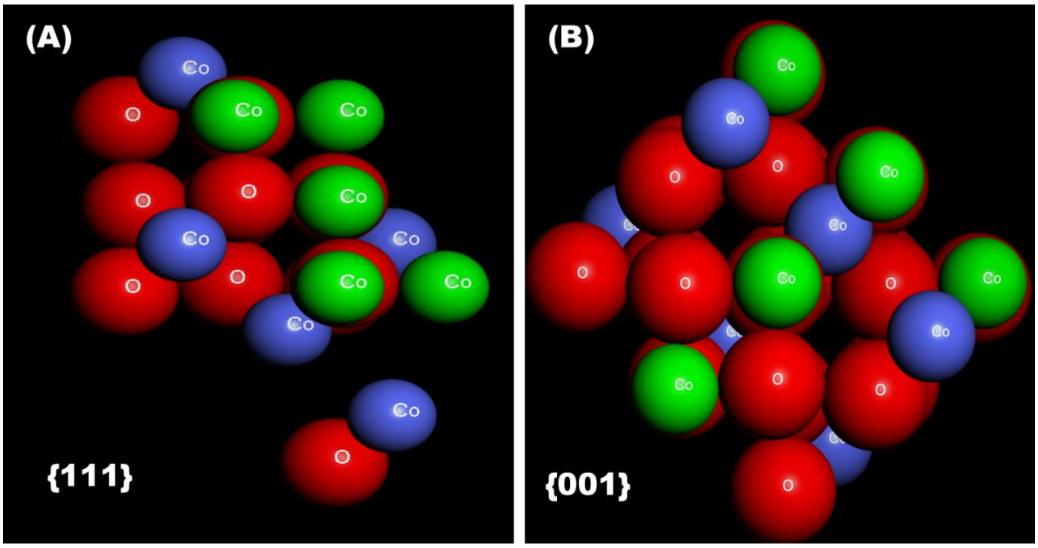

Fig. S21. (A–B) DFT models highlighting the atomic surface density of the catalytic sites after multiple reuse/cycles along the projections {111} (B) and {001} (B).

References

1. Hummers, W. S., & Offeman, R. E. Preparation of graphitic oxide. *J. Am. Chem. Soc.* **80**, 1339-1339 (1958).
2. Delley, B. An All-Electron Numerical Method for Solving the Local Density Functional for Polyatomic Molecules. *J. Chem. Phys.* **92**, 508–517 (1990).
3. Delley, B. From molecules to solids with the DMol3 approach. *J. Chem. Phys.* **113**, 7756–7764 (2000).
4. Perdew, J. P., Burke, K., & Ernzerhof, M. Generalized gradient approximation made simple. *Physical Review Letters*, **77,** 3865–3868 (1996).
5. Delley, B. Hardness conserving semilocal pseudopotentials. *Phys. Rev. B* **66**, 155125 (pp.1-9) (2002).
6. Qiu, Y., Zhiyi, L., Xiaoming, S., & Junfeng, L. Ultrathin Co_3O_4 nanosheet arrays with high supercapacitive performance. *Sci. Rep.* **3** , 3537 (2013).
7. Dezhi, K. Chuanwei, C., Ye, W., Jen, I.W., Yaping, Y., & Hui, Y. Y. Three-dimensional Co_3O_4@C@Ni_3S_2 sandwich structured nanoneedle arrays: towards high performance flexible all-solid-state asymmetric supercapacitors. *J. Mater. Chem. A* **3**, 16150–16161 (2015).
8. Ashok, K. D., Rama, K. L., Nam, H. K., Daeseung, J., & Joong, H. L. Reduced graphene oxide (RGO)-supported $NiCo_2O_4$ nanoparticles: an electrocatalyst for methanol oxidation. *Nanoscale* **6**, 10657–10665 (2014).
9. Huanlei, W., Nan, M., Jing, S., Qigang, W., Wenhua, Y., & Xin, W. Cobalt oxide-carbon nanosheet nanoarchitecture as an anode for high-performance lithium-ion battery. *ACS Appl. Mater. Interfaces* **7**, 2882−2890 (2015).
10. Shuang, Q. C., & Yong, W. Microwave-assisted synthesis of a Co_3O_4–graphene sheet-on-sheet nanocomposite as a superior anode material for Li-ion batteries. *J. Mater. Chem*. **20**, 9735–9739 (2010).
11. Dezhi, K., Jingshan, L., Yanlong, W., Weina, R., Ting, Y., Yongsong, L., Yaping, Y., & Chuanwei, C. Three-dimensional Co_3O_4@MnO_2 hierarchical nanoneedle arrays: morphology control and electrochemical energy storage. *Adv. Funct. Mater.* **24**, 3815–3826 (2014).
12. Deyang, Z., Hailong, Y., Yang, L., Kangwen, Q., Chunlei, W., Yihe, Z., Xianming, L., Jingshan, L., & Yongsong, L. $NiCo_2O_4$ nanostructure materials: morphology control and electrochemical energy storage. *Dalton Trans*. **43**, 15887–15897 (2014).

13. Li, C., Juan, L., & Xiao, G. Z. Preparation and properties of Co_3O_4 nanorods as supercapacitor material. *J Appl Electrochem* **39**, 1871–1876 (2009).
14. Xin, h. X., Jiang, P. T., Yong, Q. Z., Yong, J. M., Xiu, L. W., Chang, D.G., & Xin, B. Z. Freestanding Co_3O_4 nanowire array for high performance supercapacitors. *RSC Advances* **2**, 1835–1841 (2012).
15. Tian, Y. M., Sheng, D., Mietek, J., & Shi, Z. Q. Metal−organic framework derived hybrid Co_3O_4-carbon porous nanowire arrays as reversible oxygen evolution electrodes. *J. Am. Chem. Soc.* **136**, 13925−13931 (2014).
16. Jian, J., Jinping, L., Ruimin, D., Xiaoxu, J., Yingying, H., Xin, L., Anzheng, H., Fei, W., Zhihong, Z., & Xintang, H. Direct synthesis of CoO porous nanowire arrays on Ti substrate and their application as lithium-ion battery electrodes. *J. Phys. Chem. C* **114**, 929–932 (2010).
17. Meher, S. K., & Rao, G. R. Ultralayered Co_3O_4 for high-performance supercapacitor applications. *J. Phys. Chem. C* **115**, 15646–15654 (2011).
18. Genqiang, Z., & Xiong, W. L. General solution growth of mesoporous $NiCo_2O_4$ nanosheets on various conductive substrates as high-performance electrodes for supercapacitors. *Adv. Mater.* **25**, 976–979 (2013).
19. Jian, J., Jinping, L., Ruimin, D., Jianhui, Z., Yuanyuan, L., Anzheng, H., Xin, L., & Xintang, H. Large-scale uniform α-$Co(OH)_2$ long nanowire arrays grown on graphite as pseudocapacitor electrodes. *ACS Appl. Mater. Interfaces* **3**, 99−103 (2011).
20. By, Y. L., Panitat, H., & Yiying, W. $Ni_xCo_{3-x}O_4$ nanowire arrays for electrocatalytic oxygen evolution. *Adv. Mater.* **22**, 1926–1929, (2010).
21. Sheng, L. X., Jun, S. C., Xiong, W. L., & Hua, C. Z. Mesoporous Co_3O_4 and CoO@C topotactically transformed from chrysanthemum-like $Co(CO_3)_{0.5}(OH)\cdot0.11H_2O$ and their lithium-storage properties. *Adv. Funct. Mater.* **22**, 861–871 (2012).
22. Nguyen, H., & El-Safty S. A. Meso- and macroporous Co_3O_4 nanorods for effective VOC gas sensors. *J. Phys. Chem. C* **115**, 8466 (2011).
23. Sing, K.S.W., Everett, D. H., Haul, R. A. W., Moscou, L., Pierotti, R. A., Rouquerol, J., & Siemieniewska, T. Reporting physisorption data for gas/solid systems with special reference to the determination of surface area and porosity. *Pure Appl. Chem.* **57**, 603- 619 (1985).
24. Ye, D., Luo, L., Ding, Y., Liu, B., & Liu, X. Fabrication of Co_3O_4 nanoparticles-decorated graphene C for determination of l-tryptophan. *Analyst* **137**, 2840−2845 (2012).
25. Park, C. S., Kim, K. S., & Park, Y. J. Carbon-sphere/Co_3O_4 nanocomposite catalysts for effective air electrode in li/air batteries. *J. Power Sources* **244**, 72−79 (2013).

26. Zhong, S. W., Wencai, R., Lei, W., Libo, G., Jinping, Z., Zongping, C., Guangmin, Z., Feng, L., & Hui, M. C. Graphene anchored with Co_3O_4 nanoparticles as anode of lithium ion batteries with enhanced reversible capacity and cyclic performance. *ACS Nano* **4**, 3187–3194 (2010).
27. Jianxiao Zhu, Lei Huang, Yuxiu Xiao, Leo Shen, Qi Chen and Wangzhou Shi, Hydrogenated CoOx nanowire@$Ni(OH)_2$ nanosheet core–shell nanostructures for high-performance asymmetric supercapacitors, *Nanoscale* **6**, 6772-(2014).
28. Ferrari, A. C. et al. Raman spectrum of graphene and graphene layers. *Phys. Rev. Lett*. **97**, 1–4 (2006).
29. Dresselhaus, M. S.; Dresselhaus, G.; Jorio, A. Universal properties and structure of carbon nanotubes. *Annu. Rev. Mater. Res*. **34**, 247−278 (2004).
30. Avramov-Ivic, M., Strbac, S., & Mitrovic, V. The electrocatalytic properties of the oxides of noble metals in the electrooxidation of methanol and formic acid. *Electrochim. Acta* **46** 3175-3180 (2001).
31. Jing, Z., Meng, C., Chuanfu, Z., & Chen, Wang. Synthesis of mesoporous $NiCo_2O_4$ fibers and their electrocatalytic activity on direct oxidation of ethanol in alkaline media, *Electrochimica Acta* **154** 70–76 (2015).
32. Jafarian, M., Mahjani, M.G., Heli, H., Gobal, F., Khajehsharifi, H., & Hamedi, M. H. A study of the electro-catalytic oxidation of methanol on a cobalt hydroxide modified glassy carbon electrode. *Electrochim. Acta* **48**, 3423–3429 (2003).
33. Fleischmann, M., Korinek, K., & Pletcher, D. Oxidation of organic compounds at a nickel anode in alkaline solution. *J. Electroanal. Chem* **31**, 31-39 (1971).
34. Reza, O., Jahan, B. R., Shahla, F. Poly(o-aminophenol) film prepared in the presence of sodium dodecyl sulfate: Application for nickel ion dispersion and the electrocatalytic oxidation of methanol and ethylene glycol. *Electrochimica Acta* **54**, 2190–2196 (2009).
35. Bard, A. J., & Faulkner, L.R. *Electrochemical Methods: Fundamentals and Applications,* second ed., (Wiley, New York, 2001).
36. Li, G., Lei, Q., Ying, L., Yanyan, W., Jing, L., Hongyan, Y., & Dan, X. Microwave-assisted synthesis of nanosphere-like $NiCo_2O_4$ consisting of porous nanosheets and its application in electro-catalytic oxidation of methanol. *Journal of Power Sources* **261**, 317-323(2014).
37. Wei, W., Qingxin, C., Yingnan, Z., Wei, Z., Xiaofeng, & W. Xiaoyang, L. Nickel foam supported mesoporous $NiCo_2O_4$ arrays with excellent methanol electro-oxidation performance. *New J. Chem.* (2015).

38. Ashok, K., D., Rama, K. L., Nam, H. K., Daeseung, J., & Joong, H. L. Reduced graphene oxide (RGO)-supported $NiCo_2O_4$ nanoparticles: an electrocatalyst for methanol oxidation. *Nanoscale* **6**, 10657–10665 (2014).
39. Rui, D., Li, Q., Mingjun, J., & Hongyu, W. Sodium dodecyl sulfate-assisted hydrothermal synthesis of mesoporous nickel cobaltite nanoparticles with enhanced catalytic activity for methanol electrooxidation. J*ournal of Power Sources* **251**, 287-295 (2014).
40. Xin, Y. Y., Xian,Z. Y., Tao, L., Yong, J., Jin, H. L., & Xing, J. H. Facile synthesis of urchin-like $NiCo_2O_4$ hollow microspheres with enhanced electrochemical properties in energy and environmentally related applications. *ACS Appl. Mater. Interfaces* **6**, 3689−3695 (2014).
41. Heinen, M., Jusys, Z., & Behm, R., J. Ethanol, acetaldehyde and acetic acid adsorption/electrooxidation on a Pt thin film electrode under continuous electrolyte flow: an in situ ATR-FTIRS flow cell study. *J. Phys. Chem. C* **114**, 9850–9864 (2010).
42. Jianping, L., Jianqing, Y., Changwei, X., San, P. J., & Yexiang, T. Kinetics of ethanol electrooxidation at Pd electrodeposited on Ti. *Electrochemistry Communications* **9,** 2334–2339 (2007).
